# Fly Ash-Based Geopolymer Composites: A Review of the Compressive Strength and Microstructure Analysis

**DOI:** 10.3390/ma15207098

**Published:** 2022-10-12

**Authors:** Shaker Qaidi, Hadee Mohammed Najm, Suhad M. Abed, Hemn U. Ahmed, Husam Al Dughaishi, Jawad Al Lawati, Mohanad Muayad Sabri, Fadi Alkhatib, Abdalrhman Milad

**Affiliations:** 1 Department of Civil Engineering, College of Engineering, University of Duhok, Duhok 42001, Iraq; 2 Department of Civil Engineering, College of Engineering, Nawroz University, Duhok 42001, Iraq; 3 Department of Civil Engineering, Zakir Husain Engineering College, Aligarh Muslim University, Aligarh 202002, India; 4 Department of Highways & Airports Engineering, College of Engineering, University of Diyala, Diyala 32001, Iraq; 5 Civil Engineering Department, College of Engineering, University of Sulaimani, Sulaimaniyah 16278, Iraq; 6 Department of Civil and Environmental Engineering, College of Engineering, University of Nizwa, Nizwa P C 616, Ad-Dakhiliyah P.O. Box 33, Oman; 7 Peter the Great St. Petersburg Polytechnic University, 195251 St. Petersburg, Russia; 8 Department of Structural Engineering, Faculty of Civil Engineering and Built Environment, Universiti Tun Hussein Onn Malaysia (UTHM), Batu Pahat 86400, Malaysia

**Keywords:** geopolymer, fly ash, carbon dioxide emission, microstructure, alumino-silicates, compressive strength, mix design

## Abstract

Geopolymer (GP) concrete is a novel construction material that can be used in place of traditional Portland cement (PC) concrete to reduce greenhouse gas emissions and effectively manage industrial waste. Fly ash (FA) has long been utilized as a key constituent in GPs, and GP technology provides an environmentally benign alternative to FA utilization. As a result, a thorough examination of GP concrete manufactured using FA as a precursor (FA-GP concrete) and employed as a replacement for conventional concrete has become crucial. According to the findings of current investigations, FA-GP concrete has equal or superior mechanical and physical characteristics compared to PC concrete. This article reviews the clean production, mix design, compressive strength (CS), and microstructure (Ms) analyses of the FA-GP concrete to collect and publish the most recent information and data on FA-GP concrete. In addition, this paper shall attempt to develop a comprehensive database based on the previous research study that expounds on the impact of substantial aspects such as physio-chemical characteristics of precursors, mixes, curing, additives, and chemical activation on the CS of FA-GP concrete. The purpose of this work is to give viewers a greater knowledge of the consequences and uses of using FA as a precursor to making effective GP concrete.

## 1. Introduction

**Figure 1:**
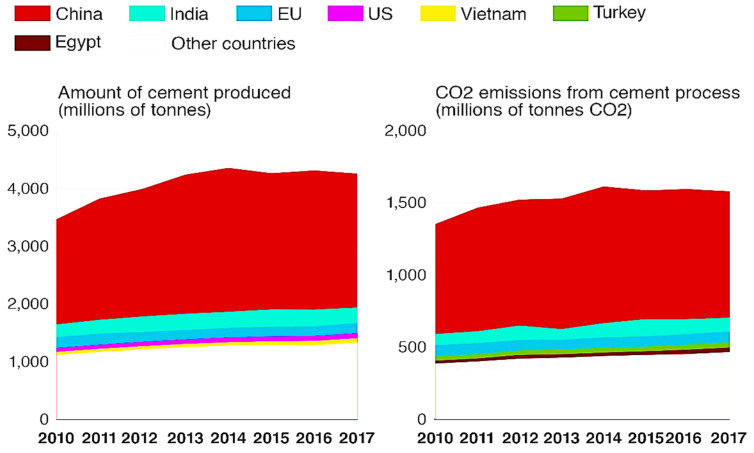
Global cement production volumes and process-related carbon dioxide emissions. Adapted from [33].

The most utilized construction material is concrete because of its performance, cost, ability to form different shapes, and availability of raw materials locally [1,2,3]. However, the increasing demand for concrete for various construction applications has led to a corresponding high consumption of raw materials utilized in the manufacture of concrete [1,4,5]. The production of PC, which is the primary binder in concrete, has also been shown to have a hugely detrimental influence on the environment. It has been estimated that approximately one ton of CO_2_ is emitted into the environment for every ton of PC produced [6,7,8,9]. It has been estimated that between 2017 to 2050, the manufacture of PC annually will grow by 50%. The substantial rise in the demand for PC to produce concrete is expected to substantially increase the amount of CO_2_ emitted into the environment. CO_2_ emissions estimated between 85 to 105 gigatons could be emitted into the atmosphere over the next 33 years because of the production of PC [6,10,11]. In addition to the high emission linked with PC production, about 2 to 3% of the worldwide energy demand is also consumed [6,11]. Figure 1 shows the global cement production and the amount of pollution associated with it. These high CO_2_ emissions ensuing from PC production would hinder the world from achieving its sustainability goals and result in more detrimental impacts on the environment. Thus, this study aims to explore alternative sustainable materials such as GP concrete that can be used in place of PC concrete [2,12].

**Figure 2:**
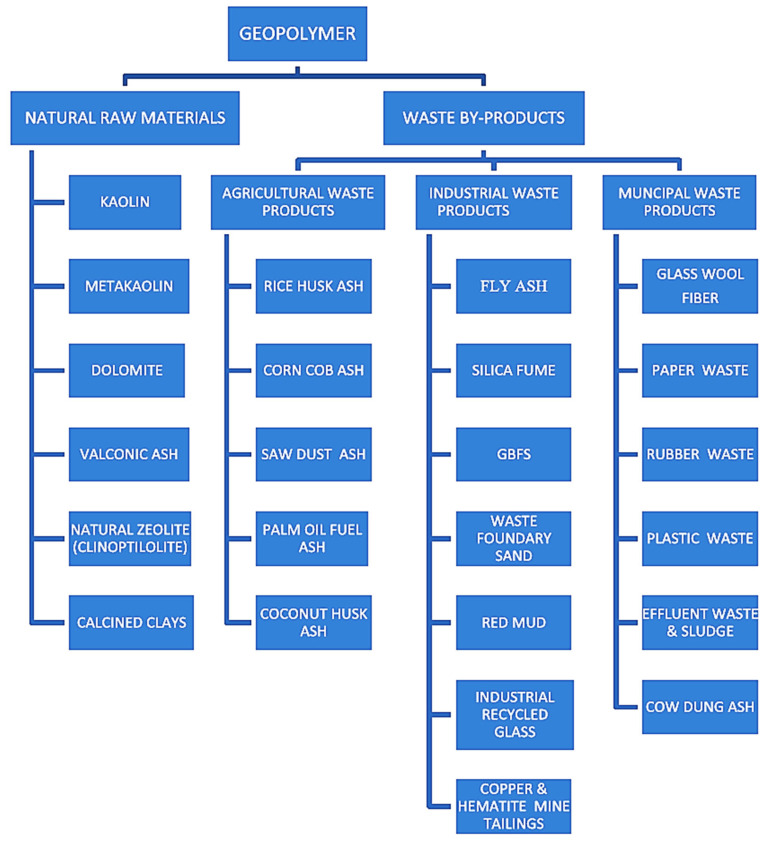
Precursor materials that are commonly utilized in GP composites. Adapted from [34].

**Figure 3:**
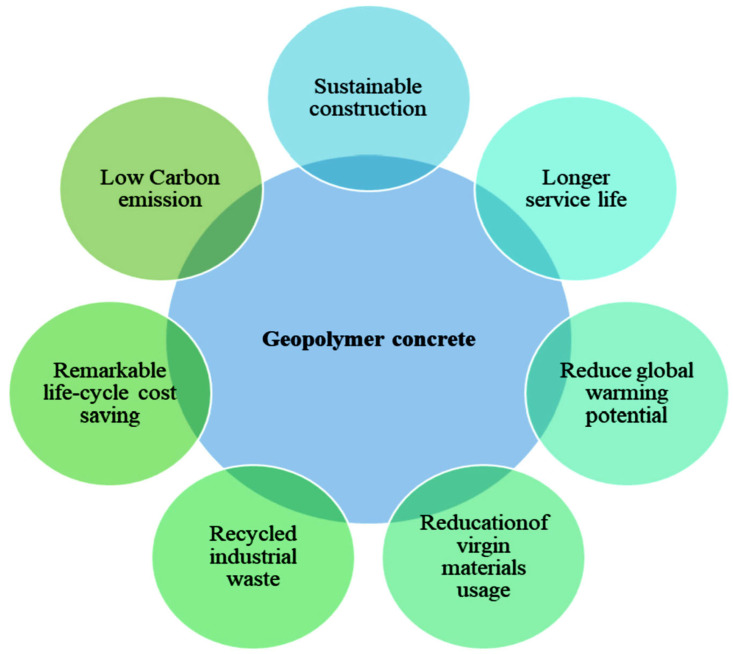
Benefits of GP concrete in sustainable construction. Adapted from [35].

GP concrete is deemed the next generation of concrete because of its sustainability benefits as it offers an innovative way to totally eliminate PC as a binder in the manufacture of concrete, thereby resulting in a substantial reduction in CO_2_ emissions [13,14,15]. Generally, in the literature, ligands with precursors based on alumina and silica are treated as GPs, while alkali-activated cement is based on precursors rich in calcium. In contrast to PC concrete, the binder phase in GP concrete is formed because of the process known as geopolymerization. Geopolymerization occurs when silicate and aluminate monomers in alumino-silicate precursors are activated with an alkali/acidic medium [16]. The alumino-silicate precursors used in GPs can be natural materials (e.g., kaolin, mining waste) or waste materials (e.g., palm oil ash, FA, rice husk ash, granulated blast slag) [17]. Figure 2 presents the common alumino-silicate precursors used in GP concrete. In addition to the sustainability benefits of GP concrete [18,19,20], the use of GP concrete also opens a pathway to effectively manage wastes that could have an adverse effect on health, safety, and the environment [21]. Studies have also shown that GP concrete with a similar cost to that of PC concrete can be produced while reducing CO_2_ emission by about 22–72% [22]. In addition, GP concrete has been shown to have superior mechanical characteristics [23,24,25,26], an adjustable thermal expansion coefficient [27], high-temperature resistance [28], and acid resistance [29,30,31,32]. Figure 3 presents a schematic diagram of using GP concrete as a sustainable construction material.

**Figure 4:**
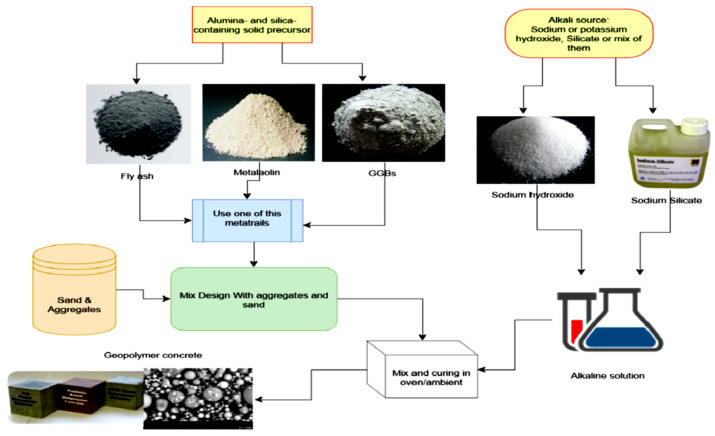
Production of GP. Adapted from [35].

GPs are composed of the alumino-silicate precursor which is composed of amorphous silica and alumina alongside an activator that acts as the dissolving agent. Geopolymerization is the process by which zeolitic-like materials are converted into a three-dimensional (3D) alumino-silicate gel [1,36,37,38]. A polycondensation procedure involving silicate and aluminate in an alkaline environment develops the corresponding performance of the generated binder [27]. The common activators used in synthesizing GPs are either MOH-type caustic alkalis or R_2_O⋅(n)SiO_2_-type silicates or both. M in the MOH indicates the alkali-metal such as K, Na, and Ca in the activator, which is generally sodium hydroxide (NaOH), sodium carbonate (NaCO_3_), potassium hydroxide (KOH), or sodium sulfate (Na_2_SO_4_) [39]. In addition to the common activators mentioned, various alternative activators such as rice husk ash and silica fume have been utilized as activator components in synthesizing GPs. Compared to the hydration reaction of PC, the water used in the synthesis of GPs only helps in producing a workable mixture and does not participate in the geopolymerization and polycondensation process [40]. Figure 4 presents a schematic diagram of GP production [41,42,43,44].

This study aims to analyze and answer a lot of worries concerning the viability of FA-GP concrete as the next-generation sustainable concrete. Despite these numerous advantages, the impact of FA on the long-term characteristics of GP concrete remains a source of worry that must be fully understood before FA-GP concrete can be considered a sustainable alternative. In a summary, the purpose of this work is to give viewers a greater knowledge of the consequences and uses of using FA as a precursor to making effective GP concrete [45,46,47].

**Figure 5:**
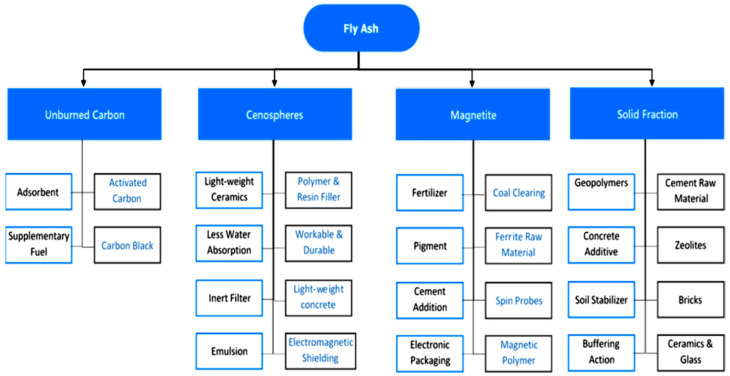
FA’s main components and applications. Adapted from [52].

**Figure 6:**
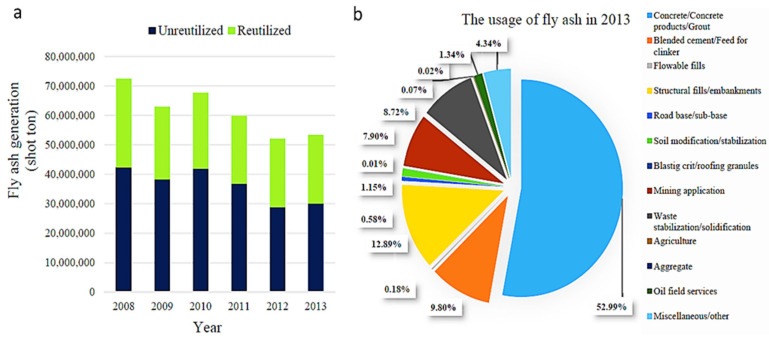
(**a**) FA generation in the United States from 2008 to 2019; (**b**) FA application fields in the United States in 2019. Adapted from [53].

Depending on their local availability, use, and production cost, various types of precursors have been used to produce GPs [5,36,48]. The most common precursor used to make GP concrete is FA because of its high composition of silicate and aluminate coupled with its local availability. FA has been extensively used in PC concrete to partially replace PC as the binder [49]. Figure 5 presents many applications of FA compounds. The global FA generation is anticipated to reach over 363 million tons per year, with India leading the way, followed by China and the US [13,22]. Figure 6 presents global FA generation and usage. As the precursor, about 450 kg/m^3^ of FA is utilized for every ton of FA-GP concrete [22]. Because FA is the primary ingredient used for the synthesis of FA-GP concrete, it is anticipated that about 2 billion tons of FA would have been needed to substitute 100% PC [22,50]. As there is a substantial quantity of FA available worldwide, its use in developing FA-GP concrete can be deemed a sustainable tactic [51]. This review paper focuses on the up-to-date development of FA-GP concrete. The emphasis is on identifying how the silicon/aluminum (S/A) ratio proportions, the additives, and the kind and quantity of alkali sol are employed to controllably enhance the qualities of FA-GP concrete. The critical property of concrete, namely the CS, is also discussed. A relationship between the Ms characteristics and CS of the FA-GP concrete is also established.

## 2. Significance of Study

This study aims to analyze and answer a lot of worries concerning the viability of FA-GP concrete as the next-generation sustainable concrete. Numerous investigators that presented evaluations on FA-GP concretes discovered that the strength characteristics of the FA-GP concrete might be equal, if not considerably higher, compared to those of PC concrete [54,55]. Furthermore, the application of FA-GP concrete technologies may give environmental and economic benefits. As a result, FA-GP concrete is growing in recognition in the construction sector. Despite these numerous advantages, the impact of FA on the long-term characteristics of GP concrete remains a source of worry that must be fully understood before FA-GP concrete can be considered a sustainable alternative. A small number of review articles have been published that provide a complete overview of the uses and effects of FA on the different technical features of GP concrete. Similarly, the current work intends to evaluate and compile the outcomes of numerous investigations under one tent, with a focus on the Ms characteristics of FA-GP concrete. This review study combines the clean manufacturing of FA particles and parameters influencing the properties of FA-GP concrete and addresses the Ms characteristics and CS of FA-GP concrete in depth. In a summary, the purpose of this work is to give viewers a greater knowledge of the consequences and uses of using FA as a precursor to making effective GP concrete

## 3. Geopolymerization

Geopolymerization is a chemical reaction between alumino-silicate precursors and an alkaline component that results in a hardened product at room temperature or occasionally at an increased temperature [56]. Alkali-activated binders or alkali-activated materials are two terms for the same thing [57]. Materials rich in silica and alumina, such as ferrous slag, non-ferrous slags, calcined clay, and natural pozzolans, can be utilized as a source of alumino-silicate in geopolymerization [58,59,60]. The suggested structure of GP by Davidovits [56] is presented in Equation (1).

Mn[(SiO_2_)Z AlO_2_]n,wH_2_O (1)

**Figure 7:**
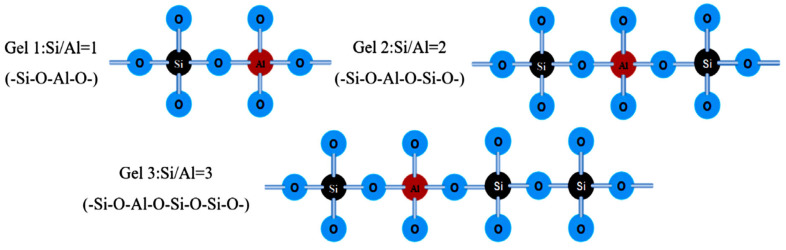
GP structures according to Davidovits [56]. Adapted from [66].

In Equation (1), M is a symbol of the alkali-metal cation, n is a symbol of the gradation of polycondensation, w is a symbol of the quantity of chemically attached water molecules, and z is a symbol of the silicon to an aluminum ratio. The GP structures are classified into three varieties based on the value of z: -Si-O-Al-O- (Gel 1, S/A = 1), -Si-O-Al-O-Si-O- (Gel 2, S/A = 2), and -Si-O-Al-O-Si-O-Si-O- (Gel 3, S/A = 3) [61,62,63]. Figure 7 illustrates these structures.

**Figure 8:**
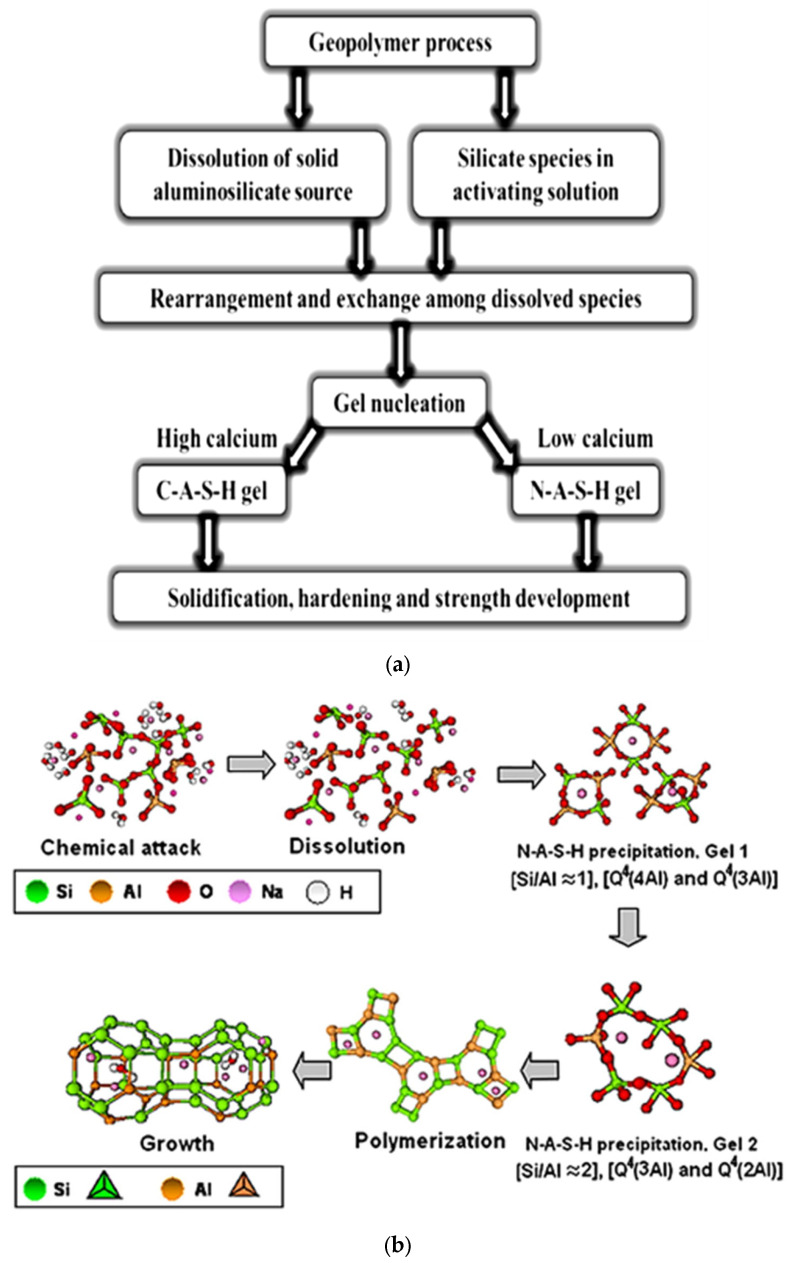
(**a**) A Provis conceptual model of geopolymerization [64,69]; (**b**) a Glukhovsky conceptual model for geopolymerization [70].

Figure 8 presents a rough idea of how a conceptual model of [64] works in the GP reaction. Dissolution of alumina and silica happens in GP reactions because of an alkaline environment composed of sodium and potassium hydroxides and silicates; the rearrangement and swapping of dissolved species results in GP gel formation [65]. Based on the chemical composition of the precursors, gel formation in GP reactions is classified into two types. Blast furnace slag (BFS) is a high-calcium precursor that creates the (C-A-S-H)– type of gel, whereas metakaolin (MK) and FA are low calcium precursors that make the N-A-S-(H) type of gel [64,65]. Strength is developed as the GP gel hardens.

In comparison, five distinct chemical processes exist for the GP, as illustrated in Figure 8b showing the Shi, Jiménez, and Palomo conceptual model [66,67,68]. When activated silico-aluminous cementitious materials react with alkaline activators, GPs are generated. According to this paradigm, polymerization processes are categorized into four groups: deconstruction, gel formation, polycondensation, and crystallization.

## 4. FA-GP Concrete

**Figure 9:**
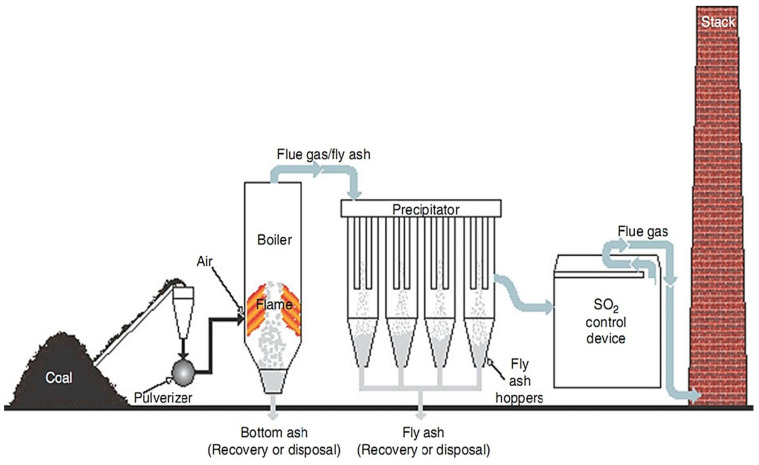
Clean production of FA. Adapted from [84].

**Figure 10:**
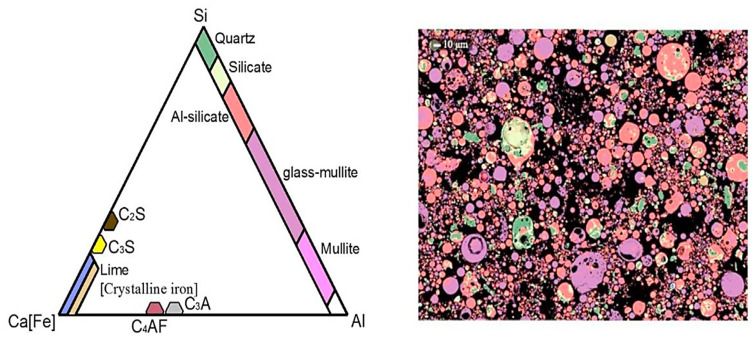
SEM-EDS characterization of FA mineral phase composites based on intuition. Adapted from [79].

FA is an abbreviation for “pulverized fuel ash” generated after the collection of boiler flue gas from coal power plants, as shown in Figure 9 [71,72]. The characteristics of the FA in terms of chemical and physical can be correlated to calcined condition and formation process. FA’s primary chemical compositions are SiO_2_, Al_2_O_3_, and CaO [73], and the amorphous slope glass dosage has the greatest influence on activity [74,75,76,77]. The noxiousness of FA leachate exhibited negligible ecotoxicity [78]. SEM-EDS analysis indicates that FA possesses mullite and quartz phases [79] as shown in Figure 10. These phases are critical to the strength development of GPs [80,81,82,83].

**Figure 11:**
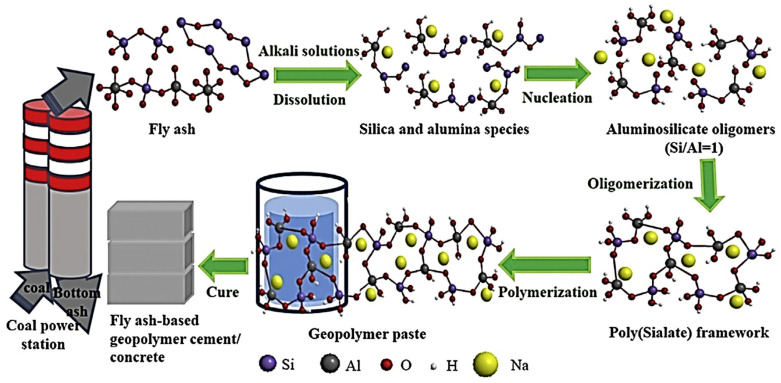
The schematic diagrams illustrate the pathway from FA to FA-GP concrete. Adapted from [90].

The formation of FA-GP concrete is based on the breakdown of alumino-silicate in FA, which is accelerated by alkali, followed by polycondensation. Manufacturing is supposed to be more energy- and resource-efficient since reactions can occur at low temperatures. The actual responses that occur during the process, on the other hand, are exceedingly complicated and remain a mystery. Condensation between the resulting Si^4+^ and Al^3+^ molecules occurs because of FA–alkali reactions, followed by more intricate nucleation, oligomerization, and polymerization, culminating in a novel alumino-silicate-based polymer with a 3D network structure. In experiments or applications, the produced FA-GP concrete is placed in a mold and dried in an oven at a specific temperature or at room temperature for a specified period to form the structure indicated in Figure 11.

Alkali activation of FA is expected to be critical for geopolymerization formation: in an alkaline sol, the silica, alumina, or alumino-silicates in FA hydrolyze, and the -Si-O-Si- or -Si-O-Al- bonds of alumino-silicate break, releasing active Al^3+^ and Si^4+^ species, which combine to form nuclei and alumino-silicate oligomers composed of SiO_4_ and AlO_4_ tetrahedra. Based on the S/A ratio, the networks in alumino-silicate oligomers can be polyciliate -Al-O-Si-chain, polyciliate siloxo -Al-O-Si-Si-chain, or polyciliate disiloxo -Al-O-Si-Si-Si-. Al^3+^ partly replaces Si^4+^ in alumino-silicate monomers, and the resulting negative charge in the alumino-silicate bonds is neutralized by alkali cations such as K^+^ and Na^+^ [85,86], as illustrated in Figure 11. In this situation, the S/A ratio substantially impacts the final structure of the resulting GP composites [87]. It has been discovered, for example, that the S/A ratio in the FA reactor has a substantial influence on the pores (number and size) of crystalline products, which is one of the major characteristics governing the mechanical strength of GP compositions [88,89]. Table 1 outlines the various FA categories and mineral phases, providing details on their possible usage as GP precursors and the prospective end product.

## 5. Chemical Composition of Binder Materials

**Figure 12:**
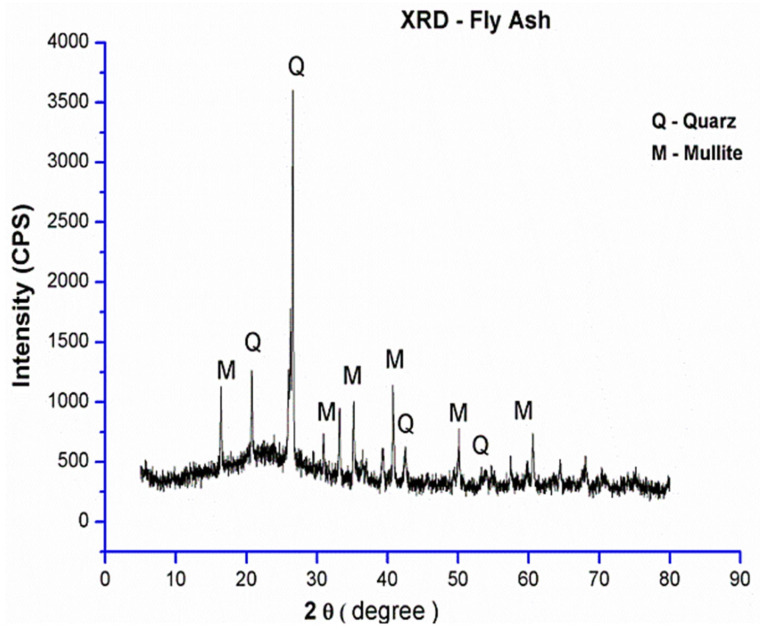
XRD of FA. Adapted from [113].

**Figure 13:**
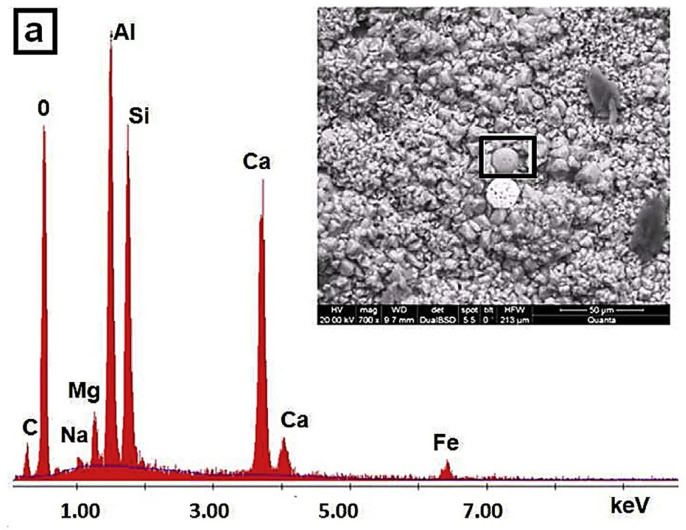
SEM and EDX curve showing the chemical composition of FA spots. Adapted from [49].

Various parameters such as particle size, S/A ratio, amorphous composition, and phase composition affect the structure and characteristics of GPs [92]. Precursors such as FA have a high amount of alumina and silica, granting them the ability to act as supplementary cementitious materials. Silicon oxide and aluminum oxide are the two most common oxides found in FA. Because PC is made from limestone, it has more calcium oxide and silicon oxide but less aluminum oxide. Table 2 presents the conclusions of XRF experiments on FA industrial by-products utilized in the production of GPs that have been described in different articles. In addition, Figure 12 shows an XRD study of several raw materials, indicating the materials’ phases. Figure 13 shows SEM of FA concrete in conjunction with EDS in the compositions of different hydration products in the same picture at multiple places.

**Table 2:** Oxide composition for PC and FA binder materials.

Material and Ref.	Composition (wt.%)															
	SiO_2_	Fe_2_O_3_	Al_2_O_3_	CaO	Pb	Na_2_O	P_2_O_5_	MnO	K_2_O	SO_3_	MgO	SrO	TiO_2_	CuO	Cl	LOI
PC [93]	19.0	3.20	4.68	66.8	26.8	0.09	0.08	0.19	1.17	3.00	0.81	-	-	-	-	2.48
FA [94]	48.8	10.20	27.00	6.20	86.0	0.37	1.20	0.15	0.85	0.22	1.40	0.16	1.30	–	–	1.70
FA [95]	61.8	4.11	28.05	0.87	94.0	0.40	-	-	0.82	1.32	0.38	-	-	-	-	0.49
FA [96]	49.0	3.00	31.00	5.00	83.0	4.00	1.00	–	1.00	0.00	3.00	–	2.00	–	–	0.00
FA [93]	50.7	8.80	28.80	2.38	88.3	0.84	–	–	2.40	0.30	1.39	–	–	–	–	3.79
FA [97]	26.4	30.13	9.25	21.6	65.7	–	0.67	0.27	2.58	1.30	–	–	3.07	–	–	3.02
FA [98]	53.5	7.47	28.80	1.55	89.7	–	–	–	–	0.14	0.81	–	–	–	–	3.11
FA [99]	64.9	5.69	26.64	0.33	97.3	0.49	–	–	0.25	0.33	0.85	–	–	–	–	0.45
FA [100]	17.5	12.43	36.37	10.5	66.3	–	–	–	1.77	1.39	3.05	–	0.88	–	–	1.19
FA [101]	54.7	5.15	27.28	5.31	87.1	0.43	1.12	0.10	1.00	1.01	1.10	0.36	1.82	0.01	–	6.80
FA [102]	27.3	2.01	50.85	5.41	80.2	0.04	–	0.02	0.33	–	0.28	–	2.12	–	–	7.74
FA [103]	50.6	6.35	18.96	14.1	75.9	0.69	–	–	–	0.74	3.12	–	–	–	–	0.17
FA [71]	57.6	5.80	28.90	0.20	92.3	–	–	–	0.90	0.20	0.90	–	–	–	–	3.60
FA [104]	63.1	3.07	24.88	2.58	91.0	0.71	0.17	0.05	2.01	0.18	0.61	–	0.96	–	–	1.45
FA [105]	66.5	3.54	22.47	1.64	92.5	0.58	–	–	1.75	0.10	0.65	–	0.88	–	–	1.66
FA [106]	51.1	12.50	25.70	4.30	89.3	0.80	0.90	0.20	0.70	0.20	1.50	–	1.30	–	–	0.60
FA [107]	47.8	14.09	28.00	3.81	89.9	0.41	1.81	0.21	0.62	0.27	0.93	–	1.99	–	–	0.43
FA [108]	54.4	8.14	27.72	1.29	90.3	0.67	–	–	–	0.11	–	–	–	–	–	4.11
FA [109]	52.7	5.92	18.05	12.9	76.7	1.11	–	0.14	2.09	1.76	3.86	–	1.01	–	–	1.60
FA [110]	58.4	4.19	23.80	7.32	86.3	1.43	–	–	1.02	0.44	1.11	–	–	–	–	0.50
FA [111]	35.8	17.31	15.05	17.1	68.2	1.58	0.30	–	3.12	5.94	2.34	–	–	–	–	0.10
FA [112]	50.8	6.82	23.15	6.87	80.8	1.29	1.14	–	2.14	1.24	1.70	0.19	1.01	–	–	0.55

## 6. Mix Design of GP Concrete

GP concrete’s CS is heavily influenced by its content and mix design. As demonstrated in Table 3, variables such as SiO_2_/Al_2_O_3_, temperature curing, supplementary water, the molarity of Na_2_O/Al_2_O_3_, and NaOH ratio have a critical influence on the CS of FA-GP concrete [114,115,116]. Pimraksa, Chindaprasirt, Rungchet, Sagoe-Crentsil, and Sato [117] observed that increasing the molarity of NaOH accelerates the geopolymerization process, leading to increased CS [118,119,120,121]. Moreover, the findings by Al Bakri, Kamarudin, Bnhussain, Nizar, Rafiza, and Zarina [122] and Songpiriyakij, Kubprasit, Jaturapitakkul, and Chindaprasirt [123] demonstrated that the Na_2_SO_3_/NaOH ratio has a substantial impact on GP concrete strength growth. Other studies [124,125,126,127,128,129,130,131,132,133] have also observed that temperature and curing time have a considerable impact on the mechanical characteristics of GP concrete.

The creation of a standard for GP concrete is currently in its early stages. June [134], Ferdous, Manalo, Khennane, and Kayali [135] and Anuradha, Sreevidya, Venkatasubramani, and Rangan [136] offered numerous strategies for achieving mixed GP concrete proportions. According to Lokuge, Wilson, Gunasekara, Law, and Setunge [137], in comparison to PC concrete, creating the design mix of GP concrete is complicated and challenging as a result of the involvement of various variables.

The low-calcium FA was used in all experimental trials indicated in Table 3. The Fe_2_O_3_ percentage is around 10 to 20% by weight, while the CaO percentage is less than 5% [95,112,138]. According to research on the particle size distribution of FA, 80% of the particles of FA were smaller than 50 µm [127].

**Figure 14:**
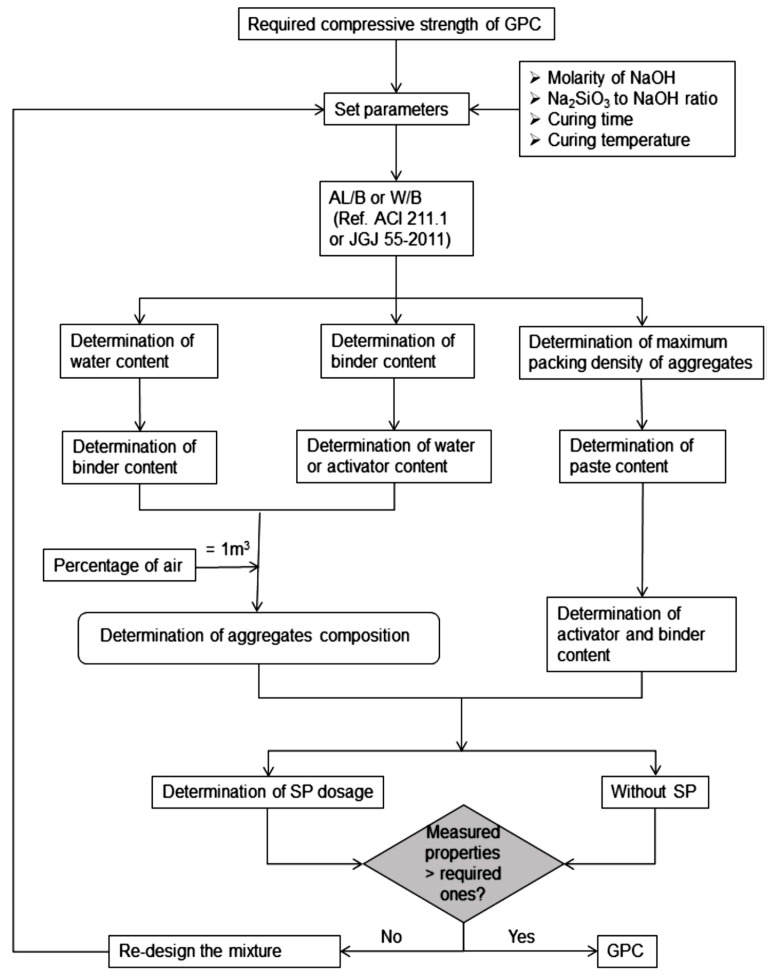
Mixture design method for GP concrete. Adapted from [141].

As in PC concrete, fine and coarse aggregates can be used as aggregates in GP concrete [126,127]. The alkali activator (AA) used to produce FA-GP concrete can be a combination of NaOH sol and Na_2_SiO_3_ sol. Aluminum oxides and silicon contribute to over 80% of the total weight, with a silicon-to-aluminum ratio of almost 2 for low-calcium FA. The binder is the primary distinction between GP concrete and PC concrete. The GP paste is made by reacting Al_2_O_3_ and SiO_2_ within FA with an alkaline activator sol, which bonds unreacted components to the GP concrete [27]. Sand and gravels, as in PC concrete, make up roughly 75 to 80% of the GP concrete mass. The technologies now available for PC concrete can compute this component of GP concrete mixes [139]. Phoo-ngernkham, Phiangphimai, Damrongwiriyanupap, Hanjitsuwan, Thumrongvut, and Chindaprasirt [140] proposed using the multivariate adaptive regression spline (MARS) approach to formulate a GP concrete combination with a 28-day goal strength. This approach is based on contour plots generated between a variety of variables that affect GP concrete’s CS. The method illustrated in Figure 14 can be utilized to build a GP concrete mix design.

The amounts and characteristics of the basic ingredients used in the creation of GP concrete influence its workability and CS. According to Pimraksa, Chindaprasirt, Rungchet, Sagoe-Crentsil, and Sato [117], increasing the dosage of NaOH sol increased the CS of GP concrete. It was also discovered that increasing the ratio of Na_2_SiO_3_ sol to NaOH sol by mass results in higher CS of GP concrete and that increasing the workability of GP concrete by incorporating superplasticizer, up to 4% FA by mass, improves the workability of GP concrete; however, this may hurt the CS of GP concrete. It was also discovered that as GP concrete’s water content increases, its workability increases.

**Table 3:** Composition of FA-GP concrete.

Ref.	FA (Kg/m^3^)	Aggregates (kg/m^3^)		Alkaline Activator (AA) (kg/m^3^)		NaOH Molarity (M)	Added Water (kg/m^3^)	Curing Conditions		Strength (MPa)
		Fine	Coarse	Na_2_SiO_3_ (SS)	NaOH (SH)			Temperature (C)	Time (h)	
[142]	450	500	1150	162	108	16	24	24	24	38.4
	450	500	1150	162	108	12	24	24	24	41.4
	450	500	1150	162	108	8	24	24	24	40.0
[143]	400	651	1209	114.3	45.7	14	22	22	24	26.7
[144]	349	620	1221		194	12	13.3	23	24	41.7
[145]	225	627	1164	112.5	45	14	24	24	24	41.1
[146]	400	548	1164	160	80		24	24	24	47.0
	300	623	1323	108	72		24	24	24	39.0
[147]	400	554	1293	113	45	14	100	100	24	33.5
[148]	400	850	950	143	57.0	12	40	70	24	53.5
	400	850	950	143	57.0	12	48	60	48	44.8
	400	850	950	143	57.0	12	60	70	24	37.3
	400	850	950	143	57.0	12	80	70	24	22.6
[90]	400	658	1222	84	56.0	14	0.0	20–23		27.0
	400	658	1222	100	40.0	14	0.0	20–23	72	25.0
[124]	408	647	1201	103	41.0	14	0.0	90	24	89.0
	408	554	1294	103	41.0	14	21.3	30	24	32.0
[142]	476	554	1294	120	48.0	14	0.0	60	24	68.0
	408	647	1201	103	41.0	8.0	0.0	60	24	63.0
	476	554	1294	120	48.0	8.0	0.0	60	24	57.0
	408	647	1201	103	55.4	8.0	0.0	75	24	44.0
	408	647	1201	103	41.0	10	7.5	60	24	45.0
	408	554	1294	103	41.0	14	0.0	60	24	44.0
	408	647	1201	103	41.0	14	17.6	60	24	43.0
	408	647	1201	103	41.0	12	14.4	60	24	42.0
	408	554	1294	103	51.5	14	16.5	60	24	42.0
	408	554	1294	103	51.5	14	16.5	60	24	41.0
	408	554	1294	103	51.5	14	16.5	60	24	41.0
	408	554	1294	103	41.0	14	16.5	60	24	40.0
	408	647	1201	103	41.0	16	26.5	60	24	40.0
	408	647	1201	103	41.0	14	20.7	90	4	37.0
	408	554	1294	103	51.5	14	16.5	60	24	36.0
	408	554	1294	103	41.0	14	10.7	30	24	35.0
	408	554	1246	103	41.0	8.0	20	60	18	29.0
	408	554	1080	103	41.0	8.0	20	60	18	29.0
	408	554	1243	103	41.0	8.0	20	60	18	25.0
[127]	408	616	1232	103	41.0	14	0.0	60	24	66.8
	408	616	1232	103	41.0	8.0	0.0	60	24	55.0
	408	616	1232	103	41.0	10	7.5	60	24	52.0
	408	616	1232	103	41.0	12	14.4	60	24	51.0
	408	616	1232	103	41.0	16	26.5	60	24	48.0
	408	616	1232	103	41.0	14	20.7	60	24	45.0
	408	616	1232	103	41.0	14	10.6	60	24	35.0
	408	616	1232	103	55.4	8.0	0.0	60	24	33.0
	408	616	1232	103	41.0	14	21.3	60	24	32.0
	408	616	1232	103	48.0	14	0.0	60	24	28.0
[94]	350	645	1200	103	41.0	8.0	35	80	24	20.0
[149]	420	750	1125	100.0	40.0	16	0.0	100	24	70.5
	400	535	1356	128.6	51.5	16	12.7	100	24	52.0
	380.	540	1233	141.3	56.5	16	14.6	100	24	49.0
	405	545	1235	132.4	52.9	16	28.0	100	24	46.0
	400	540	1265	105.7	42.3	16	24.3	100	24	44.0
	309	648	1204	59.0	27.7	10	83.7	100	24	42.0
	254	694	1290	48.5	22.8	10	68.7	100	24	36.8
	365	602	1118	73.0	34.3	10	103.5	100	24	35.3
[150]	408	554	1294	103	41.0	14	22.5	60	24	36.0
	350	645	1200	103	41.0	8.0	35	60	24	48.0
[134]	400	850	950	144	57.0	12	48	60	24–96	48.5
	408	554	1294	103	41.0	8.0	0.0	60	24–96	56.0
[151]	428	623	1177	102.9	68.6	14	28.5	20–23	72	28.6
[152]	400	651	1209	114.3	45.7	12	0.0	60–90	24–96	26.0
[125]	406	643	1194	102.0	41.0	14	26.8	70	12	37.0
	424	623	1177	91.0	36.4	14	16.0	70	12	54.9
	462	599	1153	132.2	52.9	14	21.2	75	24	49.6
	461	623	1177	92.3	46.2	14	18.6	75	24	42.5
	498	599	1153	89.7	59.8	14	26.5	60	24	39.9
	444	623	1177	111.1	44.4	14	18.6	60	24	38.7
	480	599	1153	112.0	56.0	14	23.7	70	12	37.1
	408	623	1177	85.9	57.2	14	24.5	75	24	35.7
	394	647	1201	105.1	52.6	14	21.5	60	24	29.7
[153]	408	554	1294	103	41.0	16	22.5	60	24	45.0
	404	640	1195	102	41.0	16	20	60	24	50.0
[154]	408	647	1201	93	62.0	14	4.0	60	4–96	32.0
	408	554	1294	103	41.0	8.0	0.0	60	4–96	58.0

## 7. Compressive Strength

FA-GP concrete CS is affected by S/A ratios, alkali sols, calcium content, additives, and curing conditions.

The dosage and type of alkaline sol affect the generation of Si^4+^ and Al^3+^ from FA during geopolymerization. A dosage of alkaline sol is typically advantageous for achieving high CS [3,142,155,156,157,158,159,160,161,162,163,164,165]; however, there is an ideal limit [166]. For instance, by raising the NaOH content from 4.5 to 16.5 M, Somna, Jaturapitakkul, Kajitvichyanukul, and Chindaprasirt [157] looked into the CS of ground FA (GFA) that had been treated at room temperature. The results showed that increasing the NaOH content from 4.5 to 9.5 M improved the CS of paste specimens. While altering the NaOH dosage levels from 9.5 to 14 M improves the CS of paste specimens, it does so to a much lesser extent. Increased alumina and silica extraction is primarily responsible for the increase in CS with increasing NaOH dosages. The CS of GFA hardened pastes begins to decline at a dosage of 16.5 M sodium hydroxide. This decrease in CS is mostly caused by excess hydroxide ions, which precipitated alumino-silicate gel at a relatively young age, resulting in the formation of weaker GPs.

To boost CS, Na_2_SiO_3_ sol is generally combined with NaOH [167]. This is because of the fact that Na_2_SiO_3_ with a high viscosity can aid in the production of GP gels, resulting in a compact final FA-GP concrete Ms. Furthermore, the activation technique affects the CS of FA-GP concrete. For instance, Rattanasak and Chindaprasirt [168] first introduced NaOH sol to FA for 10 min to dissolve the Si^4+^ and Al^3+^ species, followed by Na_2_SiO_3_ for one minute to assist in generating a homogeneous GP paste. The strength of FA-GP concrete increased as a result of the independent activation. Table 3 shows the specifics of the various alkaline sols as well as the molarity of the FA-GP concrete. 

**Figure 15:**
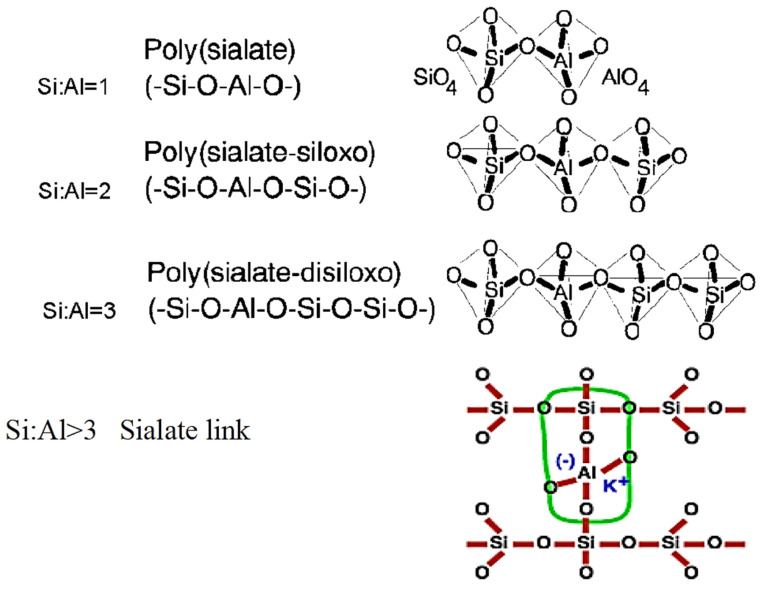
Formation of different aluminum silicate chains in alumino-silicate oligomers depending on the molar S/A ratio, which then forms the GP. Adapted from [85].

The FA-GP concrete structure is mostly determined by the S/A ratio generated by the dissolving of Al^3+^ and Si^4+^. Furthermore, the efficient S/A ratios have a substantial impact on the dissolving, hydrolysis, and condensation reactions of GPs. Condensation action in a high S/A system leads to the predominance of the silicate species itself, generating oligomeric silicates, which condense with Al(OH_4_)_4_ and create GP structures of poly(sialate-disiloxo) (Si–O–Al–O–Si–O–Si–O–) and poly(sialate-siloxo) (Si–O–Al–O–Si–O) as shown in Figure 15 [169,170]. As a result, a completely dense GP structural matrix has greater CS. Using a larger dosage of an alkaline activator, in contrast, can assist rapidity in the polycondensation of alumino-silicate oligomers by the transfer of Al^3+^ and Si^4+^ [90,171,172,173,174].

**Figure 16:**
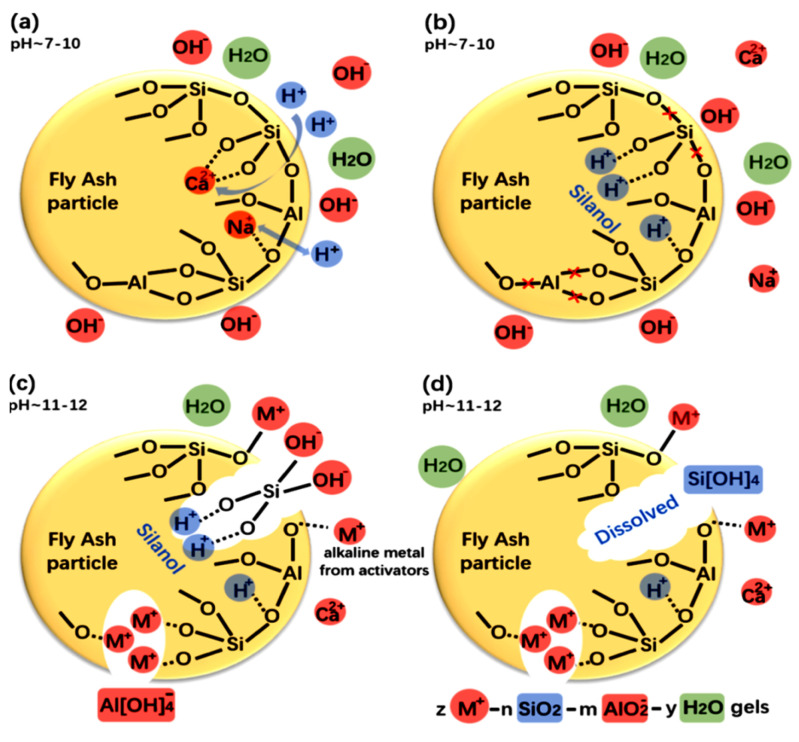
The process of alumino-silicate glass dissolving during the initial stages of reactivity of a one-part GP blend. (**a**) H^+^ exchange for Ca^2+^ and Na^+^, (**b**) dissolution of Al–O–Si bonds, (**c**) depolymerization of the glass network, and (**d**) Si and Al release. Adapted from [206,207].

The function of additives is to create a crucial equilibrium in oxide dosages to the required level using various add-ons [175,176,177,178]. Binary, ternary, and quaternary GPs have been created to achieve higher characteristics and erase any defects related to any original source [179]. For instance, Yang, Yao, Zhang, and Wang [180] found that the high calcium dosage of GBFS in FA/GBFS GP increased the initial setting time and slowed the degree of polymerization. It has been discovered that increasing GGBFS as a partial substitute for FA considerably improves both 28-day strength and short-term strength [143]. Because of the presence of C-S-H gel, iron slags as a substitute for FA in FA-GP concrete demonstrated enhanced strength with increasing slag percentage [181,182,183]. Kusbiantoro, Nuruddin, Shafiq, and Qazi [150] showed that samples cured in an oven had the maximum CS because of the polarization of OH ions, which tends to increase the pH of the sol and thus promotes the synthesis of -Si-O-Si- bonds in the GP gel (N-A-S-H gel) and CS [184,185,186,187]. While analyzing nano-structural changes in several GP composites (GBFS-based, FA-based, and mixed GBFS + FA) before and after carbonation, Bernal, Provis, Walkley, San Nicolas, Gehman, Brice, Kilcullen, Duxson, and van Deventer [188] revealed that N-A-S-H gel is the main product in FA-GP concrete that is not dissolved by carbonation and instead only alkali aluminosilicate (AAS) in the pores is impacted by CO_2_. The use of nano-alumina and nano-silica as additives in FA-GP has been found to improve its mechanical characteristics. Phoo-ngernkham, Chindaprasirt, Sata, Hanjitsuwan, and Hatanaka [176] created FA-GP pastes by adding 1% to 3% nano-silica and nano-alumina as an ingredient. The results indicated that nanoparticle additions, independent of dose, enhanced CS. This behavior was attributed to both the increased formation of C-S-H or C-A-S-H and N-A-S-H gels in the GP paste and the micro packing effect. Table 4 illustrates the effect of various additives on the behavior of GP composites. Because the dissolution and condensation of FA comprise both alkali and alkali metals, the pH of the pore sol and the alkali-metal cations are crucial for the hydration process of FA and consequently the CS. Bhagath Singh and Subramaniam [189] and Yang and Gupta [190] found that the CS of FA-GP concrete rose as alkali dosage rose, and they ascribed this to the rapid dissolution of FA at higher pH. Because network modifiers (Ca_2_^+^, Na^+^, and K^+^) are present, reacting phases including Al and Si have disorganized structures and can generate acidic -Si-OH (silanol) groups with water. The dissolution behavior of vitreous aluminum silicate-containing network modifiers is depicted in Figure 16. The presence of hydroxyl ions (OH-) in alkali sols causes the reactive alumino-silicates of FA to disintegrate. The dissolution rates of alumino-silicates are affected by their exterior and interior surface areas, as well as the degree of cross-linking in their structure [191].

**Figure 17:**
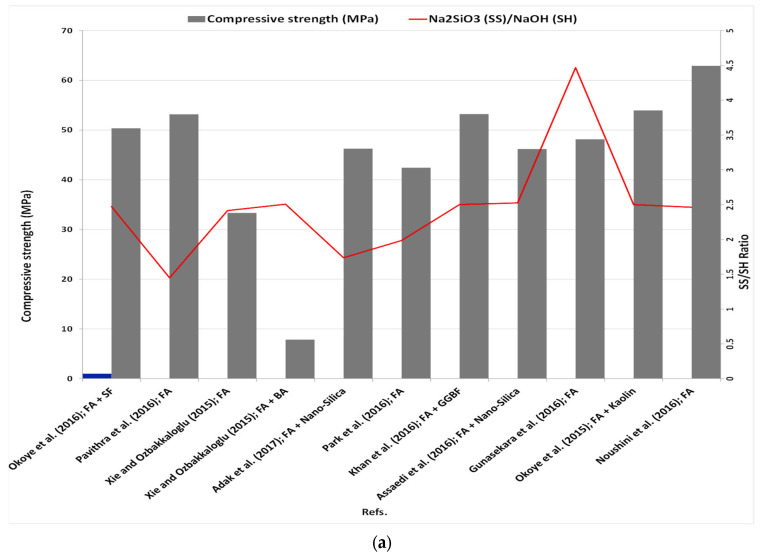

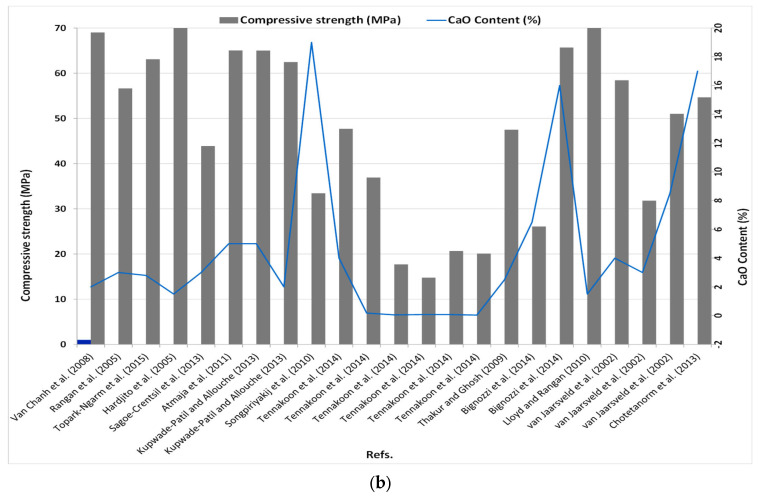
Historical statistics on CS variations in comparison to: (**a**) Na_2_SiO_3_ (SS)/NaOH (SH) [93,95,96,99,103,104,105,107,208] and (**b**) CaO content in FA-GP [123,209,210,211,212,213,214,215,216,217,218,219,220,221].

Calcium has been shown to interact with silica and alumina gelation during the geopolymerization reaction, altering the Ms of FA-GP concrete and hence the CS [192,193]. The presence of C-S-H gel and N-A-S-H gel together typically increases the CS of final products. One of the explanations for this is that the crystalline C-S-H gel reduces porosity [194,195]. Figure 17a depicts the fluctuation in FA-GP concrete CS after 28 days in relation to the Na_2_SiO_3_/NaOH (SS/SH) ratio dosage in the FA precursor.

Figure 17b depicts the change in 28-day CS of FA-GP concrete vs. CaO dosage in the FA precursor.

**Figure 18:**
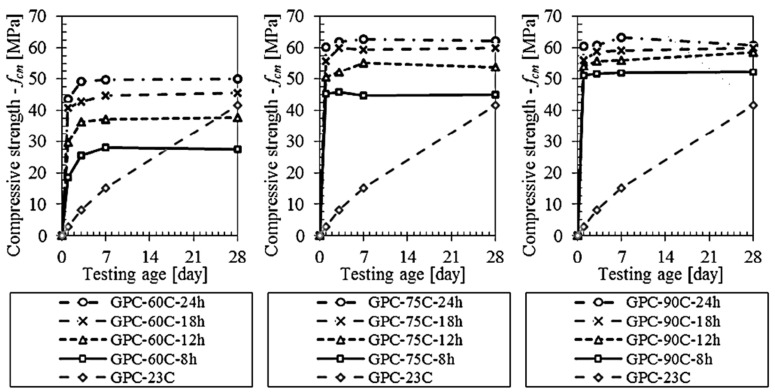
Compressive strength development of ambient and heat-cured GP concretes. Adapted from [144].

It is well established that typical GPs require thermal treatment to produce CSs equivalent to or greater than those of PC concrete [39,101,196,197,198]. Heat treatment promotes alumino-silicate gel dissolution and geopolymerization, resulting in a considerable increase in early strength [199]. Additionally, it accelerates the dissolution of silica and alumina species and the subsequent polycondensation phase. However, the thermal curing regime adopted must be suitable for promoting the proper dissolution and precipitation of dissolved silica and alumina species. Depending on the source, geopolymerization may be harmed by exceeding a specified temperature and heat treatment period, which may negatively affect the GP concrete’s mechanical properties. [156,196,200]. Noushini, Castel, Aldred, and Rawal [144] used 12 different thermal curing techniques to make FA-GP concrete with a low calcium content. Figure 18 depicts the CS of GP concrete specimens after 28 days at room temperature and after heat curing. Compressive strength initially increased faster with increasing temperature and time in cured samples, and the optimal CS was obtained at 75 °C [144]. This could be because higher temperatures and longer curing times resulted in the formation of more reactive species; hence, with the appropriate curing temperature, more metals were incorporated into the GP matrix, and metal dosages decreased [201,202,203]. Table 5 illustrates the effect of various curing regimes on the performance of GP composites.

**Figure 19:**
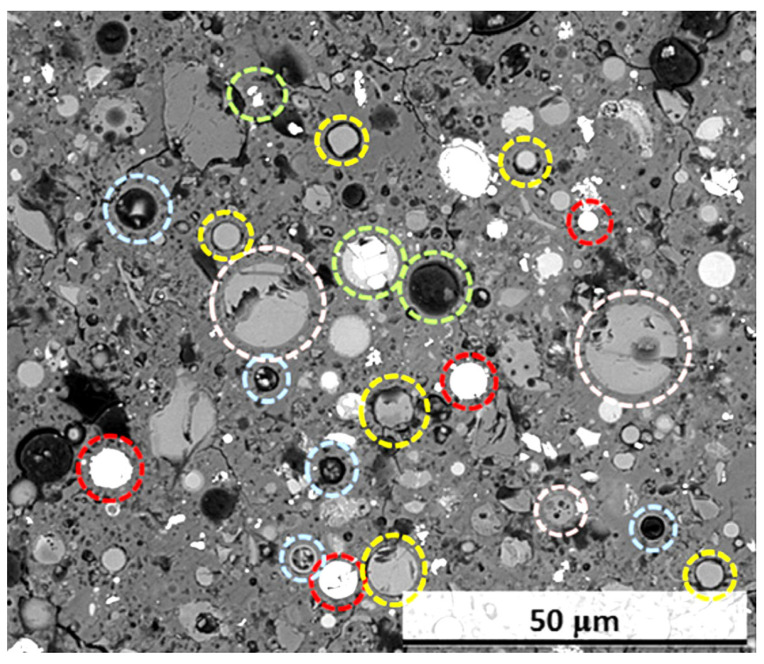
SEM image of a refined segment of an FA-GP concrete with several FA particle types highlighted in various colors. Yellow particles are reactive glass particles surrounded by a reaction rim [222,223,224]; red particles are iron-rich; pink particles are relatively unreactive glass particles with no visible reaction rim; blue particles are hollow particles whose shell was not broken during the reaction process; green particles contain visible crystalline inclusions. Adapted from [225].

The type and fineness of the GP raw material are instrumental in developing the strength, durability, and Ms of the resulting GP matrices. Figure 19 depicts an SEM image of a polished part of an FA-GP with various FA particle types. Numerous researchers have concurred that altering the particle size distribution in the composite material has a substantial effect on the CS, physical properties, and Ms of the resulting GP paste [196,204]. In general, binder phases with a finer particle size distribution have a stronger reaction and, as a result, create GP paste with a denser Ms, better CS, and improved physical properties [200,205]. Table 6 shows how particle size distribution affects the performance of GP composites.

**Table 4:** Effect of different additives on FA-GP concrete CS.

Ref.	Main Source Material	Additives	Remarks
[226]	FA	CAC	Increases strength by providing an additional supply of Al and Ca.
[227]	FA	Water glass	Increased CS because of higher Si dosage. The recommended dose is 15 g/100 mL.
[228]	FA	GBFS	With an M ratio of 0.96 SiO_2_/Na_2_O and raw materials of 70% GGBFS and 30% FA, CSs of 48 MPa were attained.
[226]	FA	GBFS	Compressive strength and Ms improve when GBFS dosage increases resulting from the production of more C-S-H.
[229]	High-calcium FA	PC	A GP composite with a more homogeneous and dense structure than concrete was created.
[197]	Low-calcium FA	GGBF, POFA	The addition of 30% POFA and 70% GGBS to FA-GP concrete produced a CS of 67 MPa. POFA dosages above 33% reduce CS.
[230]	FA	Superplasticizer	A high superplasticizer dose increases CS.
[231]	FA	Chitosan biopolymer	The introduction of N-carboxymethyl chitosan substantially improved tensile strength and resulted in a minor increase in CS.
[180]	FA	GGBFS	Slag inclusion in the raw material can improve the CS of GP mixtures, with a slag/FA dosage ratio of 0.8, providing the maximum strength.
[232]	FA	Sulfate of calcium and sodium, calcium chloride, and sucrose	Sucrose substantially slows down the ultimate setting time. All admixtures help to improve CS.
[233]	FA	GBFS, GCS	GCS partial replacement resulted in higher CS than GBFS partial replacement.
[234]	FA	Red mud	After 120 days, there is a decrease in CS. Localization of heavy metals within permitted levels.
[150]	FA	Incinerated rice husk ash	At an optimal dosage of 7%, addition resulted in an increase in compressive and bond strength.
[235]	FA	Nano-silica	The use of 6.0% nano-silica as a substitute caused improved mechanical properties.
[236]	FA	Aluminum-rich waste	A 2.5% admixture of dried powdered and calcined aluminum-rich waste gave early high strength of 34 MPa, but the CS dropped after that.

CAC, calcium aluminate cement; POFA, palm oil fuel ash; GCS, ground corex slag; GGBFS, ground granulated BFS.

**Table 5:** Curing regime and its influence on FA-GP concrete CS from different precursors.

Ref.	Precursor	Curing Regime	Remarks
[237]	FA	Curing temperatures between 66 °C and 85 °C	Curing at 85 °C for 24 h resulted in much greater strength than curing at 66 °C. Over time, the rate of growth in strength dropped after 24 h.
[167]	Class C FA	I: 62 °C for 24 h; II: 23 °C at room temperature.	The early strength of HCGC is influenced by higher curing temperatures. Compressive strength increases rapidly over the first 7 days and then gradually increases for the next 28 days.
[212]	FA/silica fume	I: 96 °C (8 h); II: 96 °C (2 h) then 150 °C (6 h) dry oven; III: 96 °C (2 h) then 96 °C (6 h) steam	50–102 MPa for I; 28–57 MPa for II. The greatest results were obtained with III: 35–77 MPa, covering with 95 °C, and stream curing.
[234]	FA slag	I: 26 °C (28 days); II: 26 °C (48 h) followed by 60 °C (4 h)	The second curing regime, which was longer in length and followed by high-temperature treatment, resulted in enhanced strength.
[167]	FA	85 °C (5 h–7 days)	Strength increases when curing time is extended. Sealed curing aids in the development of strength while also preventing early carbonation.
[238]	FA	26 °C (16 h–672 h); 41 °C (72 h) to 336 h; 60 °C (16 h–120 h); and 85 °C (1 h–6 h).	85 °C for 6 h is equivalent to 25 °C for 100 h. The strength of the K-based GP was reduced as the curing temperature increased.
[239]	FA	I: 21 days at 20 °C with an initial cure at 70 °C; II: 24 h at 20 °C with an initial cure at 70 °C.	Technique II has a higher CS than method I.
[220]	85% FA and 15% kaolin	6 h, 12 h, 24 h, and 48 h for temperatures of 30 °C, 50 °C, and 70 °C.	Curing at a greater temperature for a brief time has a positive influence on strength (a couple of hours). Structure deterioration with prolonged exposure to high temperatures.
[240]	FA	24 h at 65 °C; 5 min in the microwave. After 66 °C curing for 3 h, 6 h, and 12 h, curing at room temp	The optimal curing time was 5 min in a microwave at 65 °C for 6 h.
[241]	FA	1 h of pre-curing followed by 24 h of curing at 25, 40, and 60 °C.	Curing at 60 °C is ideal (for 7- and 28-day strength)
[200]	FA	24 h, followed by 36 h in the oven (50–90 °C).	Curing in an oven at 80 °C is ideal.
[242]	FA	Room temperature for 9–12 h, then salt water, normal water, and sealed curing environment.	The sealed state produced the best outcomes, followed by salt water and the least effective water cure.

HCGC, high calcium FA-GP concrete.

**Table 6:** Impact of the particle size distribution of precursors on FA-GP concrete CS.

Ref.	Precursors	Precursor Gradation	Main Constants	Compressive Strength (MPa)	Remarks
[243]	FA	100% GFA (1.45 m^2^/g)	Na_2_SiO_3_ with a silicate modulus of 2.5.	6.79	With the addition of finer binder content, the CS of 28 days is increased.
		25% OFA (0.395 m^2^/g) + 75% GFA		4.65	
		50% GFA and 50% OFA		3.85	
		25% GFA and 75% OFA		3.84	
[244]	FA	365 m^2^/kg	NaOH dosage of 13 M, Na_2_SiO_3_/NaOH ratio of 3, Sol/FA ratio of 0.35, curing time of 7 h at 110 °C	21.85	With increasing fineness, there is a substantial rise in CS after 28 days.
		440 m^2^/kg		29.7	
		612 m^2^/kg		42.29	
[245]	FA	d_50_ = 32.24 µm and fineness 2110 cm^2^/g 525 m^2^/kg	Alkali sol with a dosage of 13 M was utilized, and the L/S ratio was 0.35 after 4 h of curing at 90 °C.	40 62	With an increase in fineness, CS and workability improve after 28 days.
		444 m^2^/kg		57.62	
		365 m^2^/kg		39.22	
[246]	FA	X_10_ = 0.12 µm, X_99_ = 3.66 µm,	Na_2_SiO_3_/NaOH ratio of 0.4 and activator to FA ratio of 0.45%.	9.5	With an increase in fineness, there is a substantial rise in the CS of mortar after 28 days.
		X_10_ = 0.08 µm, X_99_ = 0.64 µm,		15.5	
		X_10_ = 0.04 µm, X_99_ = 0.37 µm,		19.3	
[200]	FA, RHBA	FA 75 µm + RHBA 90 µm	Curing at 80 °C for 36 h with a NaOH dosage of 12 M.	34	With an increase in fineness, there is a substantial rise in the 28-day CS of mortar.
		FA 75 µm + RHBA 7 µm		44	
		FA 3 µm + RHBA 90 µm		52.6	
		FA 3 µm + RHBA 7 µm		59.1	
[205]	FA, RHA	5.1% retained on sieve 325 of RHA	As-received FA, Na_2_SO_3_/NaOH ratio = 4, 60 °C for 48 h.	34.5	Increased fineness resulted in an overall enhancement in CS after 28 days.
		1.1% retained on sieve 325 of RHA		43	
[204]	BA	d_50_ = 15.8µm and fineness 5100 cm^2^/g	Curing at 75 °C for 48 h with a Na_2_SO_3_/NaOH ratio of 1.5 and a NaOH dosage of 10 M.	64.5	With a rise in fineness, the CS of mortar increases in 28 days.
		d_50_ = 24.4 µm and fineness 3500 cm^2^/g		48	
[158]	BA	Particle size of 15.7 µm	Liquid alkaline/ash ratios of 0.429–0.709, Na_2_SiO_3_/NaOH ratios of 0.67–1.5, and 7.5–12.5 M NaOH.	24–58	Grinding boosts reactivity and lowers porosity in BA particles, resulting in reasonably high workability and CS of 28 days.

GFA, ground FA; OFA, oil FA; RHA, rice husk ash; RHBA, rice husk-bark ash; BA, bottom ash.

## 8. Microstructure (Ms)

### 8.1. Scanning Electron Microscopy (SEM)

**Figure 20:**
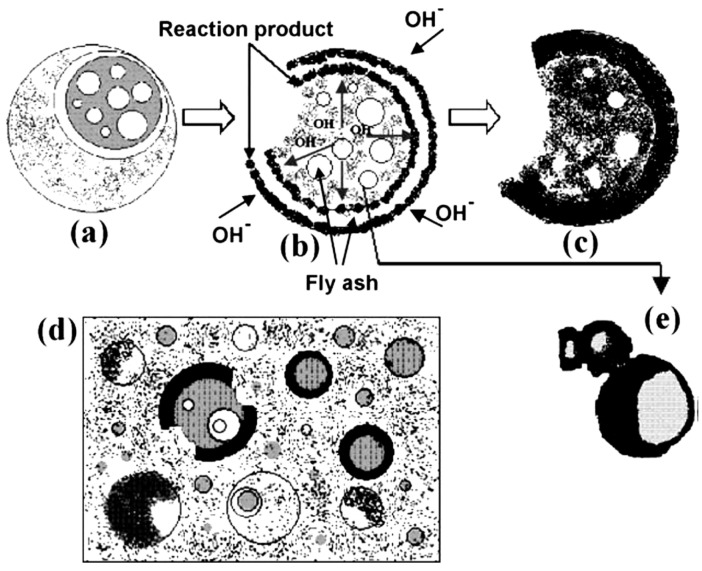
A descriptive model of FA activation by alkali: (**a**) initial chemical attack, (**b**) bi-directional alkaline attack, (**c**) reaction product, (**d**) gel, (**e**) unreacted particles. Adapted from [247].

The Ms characteristics of FA-GP concrete rely on the FA characteristics, type, and dosage of activators and the curing conditions [95,96,98]. Fernández-Jiménez, Palomo, and Criado [247] presented an explanatory model of FA-GP concrete as presented in Figure 20. Before defining the alkaline activator sol, Pavithra, Srinivasula Reddy, Dinakar, Hanumantha Rao, Satpathy, and Mohanty [95] established several fixed parameters, including dosage, SS/SH ratio, curing temperature, and duration (AAS). This study discovered that as the ratio of AAS to binder increases, the strength of GP concrete decreases because of the increased amount of water in the GP mix [16]. The contact area for the reaction was obstructed by the water molecules, and this, in turn, altered the geopolymerization process between the binder and the activator, resulting in a low compressive strength of the GP concrete [248]. When AAS/binder ratios of 0.4 and 0.5 were employed, the GP concrete matrix included a negligible percentage of unreacted FA particles. Nonetheless, it has been demonstrated that the presence of unreacted FA particles in the FOC matrix aids in matrix densification and minimizes the formation of microcracks. This behavior is consistent with other studies demonstrating that the ability of unreacted FA particles to densify the Ms of GP concrete improves strength performance [96,98,249,250]. 

**Figure 21:**
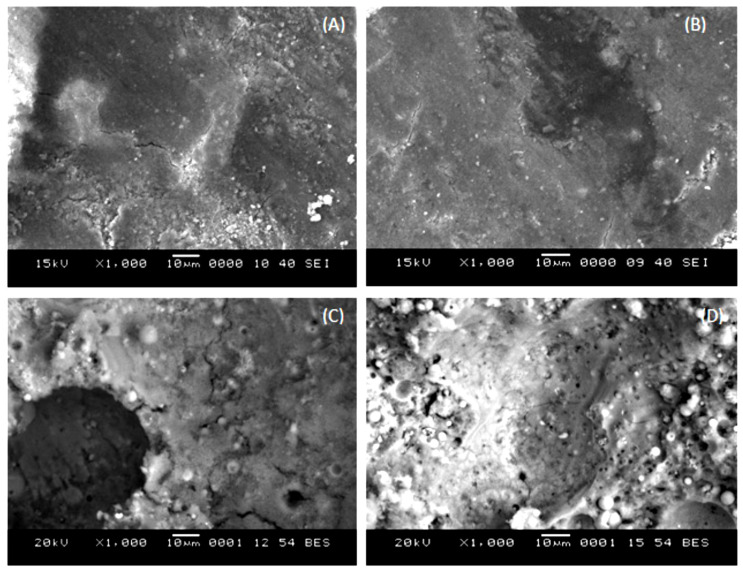
SEM images of FA-GP concrete generated at various Na_2_SiO_3_/NaOH weight proportions at 28 days: (**A**) 0.5, (**B**) 1, (**C**) 2, and (**D**) 3. Adapted from [251].

The influence of several SS to SH ratios on Ms studies has been investigated [251]. As shown in Figure 21, fully reacted FA particles were evident in GP concrete when an SS to SH ratio in the range of 0.5 to 1.0 was used. Despite the similarity in the Ms of GP concrete made with SS to SH ratios of 0.5 and 1.0, the resulting CS differs. The CS of GP concrete made with an SS to SH ratio of 1 was 65 MPa, while the GP concrete made with an SS to SH ratio of 0.5 exhibited a CS of 35 MPa for the same age. These strength findings are in agreement with a previous study where a higher dosage of silicate was found to result in better strength development as a result of the availability of additional silicates available for geopolymerization [237]. However, the Ms images presented in Figure 21 show that GP concretes made with a higher SS to SH ratio have more presence of unreacted FA particles. It is well established that an excess of AAS in the mix hampers geopolymerization by restricting the interaction of the binder and AAS [252]. When current research on this subject is compared, it is clear that most writers utilized a mass ratio of SS/SH of up to 2.5 [93,96,208,253].

**Figure 22:**
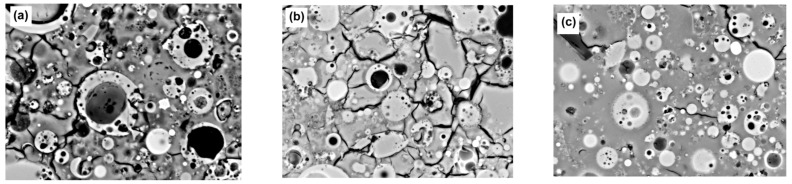
SEM images showing (**a**) pure GP combined with GP nanocomposites comprising 3.0 wt% nano-silica, (**b**) wet mix procedure, and (**c**) dry mix procedure. Adapted from [104].

Karthik, Sudalaimani, Vijayakumar, and Saravanakumar [254] included bio-additives in GP concrete and established a correlation between the Ms density and CS. It was discovered that increasing the density of the GP concrete Ms results in increased CS. These findings corroborate previous research on including nano-silica, silica fume, nano-aluminate, and graphene oxide in FA-GP concrete [255,256]. Figure 22 shows SEM images of GP concrete nanocomposites containing 3.0 wt% nano-silica in wet and dry mix conditions. The amount of unreacted particles has been substantially reduced. Additionally, the dry mix process detects fewer microcracks than the pure GP and wet mix procedures. Further, Cai, Pan, Li, Tan, and Li [257] report that FA-GP concrete has a higher electrical resistivity than metakaolin-based GP concrete.

**Figure 23:**
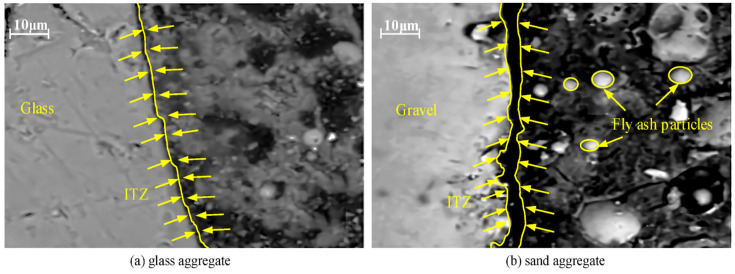
FA-GP concrete SEM images with several types of aggregates: (**a**) the interface of the glass aggregate and GP; (**b**) the interface of the sand aggregate and GP. Adapted from [258,259].

Figure 23 shows the interfacial transition zone (ITZ) in GP concrete. According to Embong, Kusbiantoro, Shafiq, and Nuruddin [100], the enhancement of FA particle dissolution and polycondensation of alumino-silicate compounds provides a link between the aggregates and the matrix. The CS of the samples was greatly increased as a result of the development of alumino-silicate filling the ITZ.

### 8.2. Wide-Angle X-ray Properties

**Figure 24:**
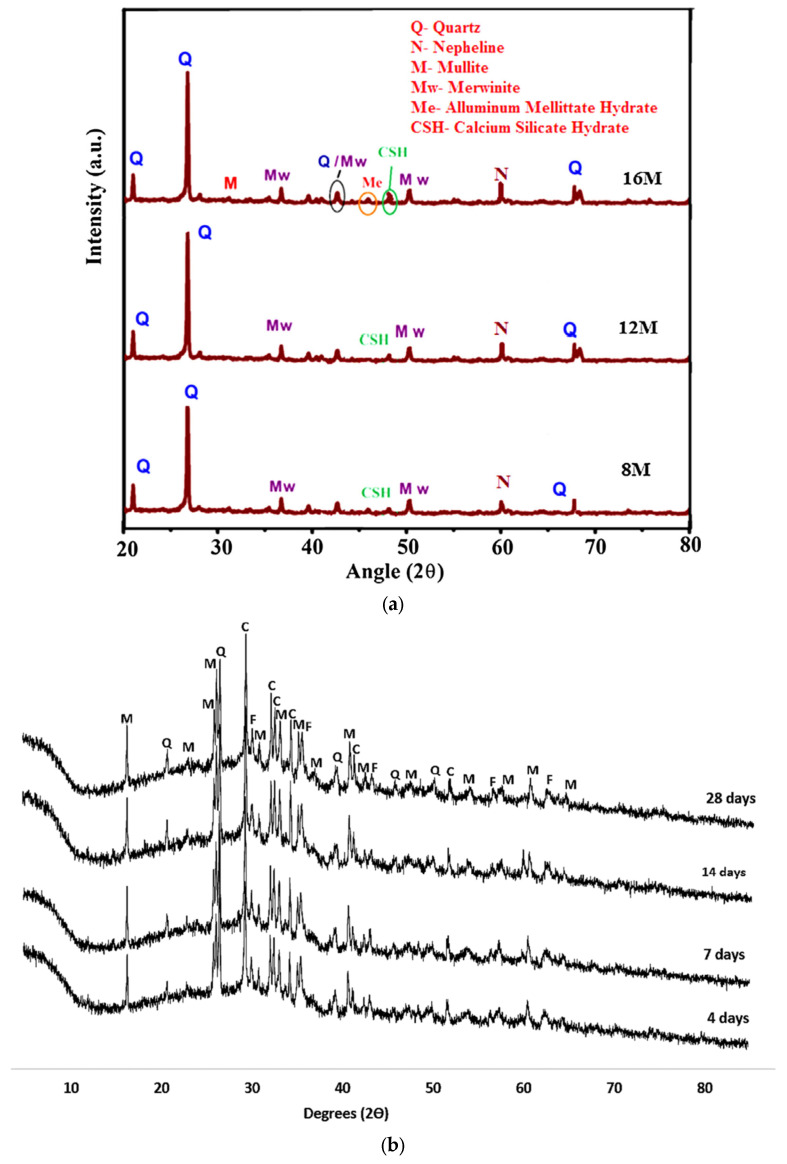
XRD pattern of FA-GP concrete specimen at different (**a**) molarities after 28 days and (**b**) ages. Adapted from [258,262].

In reality, Lloyd, Provis, and van Deventer [260] chronicled that the boost in the quantity of silica encourages the diffusion of alkalis. Nevertheless, these phase modifications taking place during aging are absent in the XRD patterns. In addition, Abdulkareem, Mustafa Al Bakri, Kamarudin, Khairul Nizar, and Saif [97] described a broad hump ranging from 20° to 35° that indicated the presence of amorphous GP products. Previous research has revealed that FA comprises quartz and mullite mineral phases, and Table 2 also shows that iron and calcium made up more than half of the FA [261]. As a result, the reason for the presence of phases in the GP paste pattern is depicted in Figure 24. Mullite Al_4_._56_Si1._44_O_9_._72_, quartz SiO_2_, magnetite Fe_3_O_4_, and calcium silicate Ca_3_SiO_5_ were recognized as crystalline phases. The inclusion of calcium silicate in the quantitative measurement of fly ash indicates slight cement contamination in Melbourne ash. The binder’s XRD phases are entirely connected to the fly ash source material, and no novel crystalline phases are generated in this binder. Furthermore, the amount of the calcium silicate phase does not decrease with time, indicating that the C3S phase in fly ash does not participate much in the reaction, and the sodium aluminosilicate hydrate (N-A-S-H) gel is still the predominant binding matrix in these specimens. As previously discussed, crystalline phases were revealed after adding an alkaline sol.

**Figure 25:**
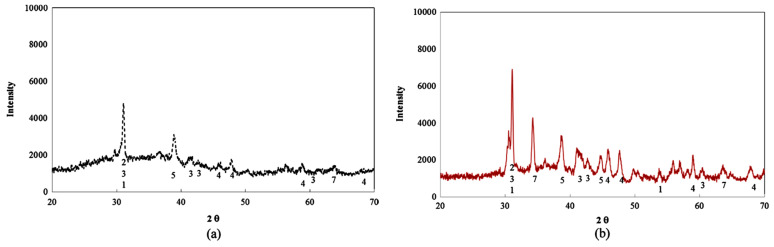
XRD spectrum (**a**) before and (**b**) after the dissolution of FA. Adapted from [108].

As shown in Figure 25, the intensity of most peaks on the XRD spectrum is weaker and a considerable amount of amorphous phases still exist after the dissolving testing. This is because the calcium content in the FA can produce fast precipitant (Portlandite) covering the surface of the FA, which not only prevents further dissolution of the FA but also reduces the intensity of the peaks of the crystalline phases [108].

### 8.3. Mercury Intrusion Porosimetry (MIP)

**Figure 26:**
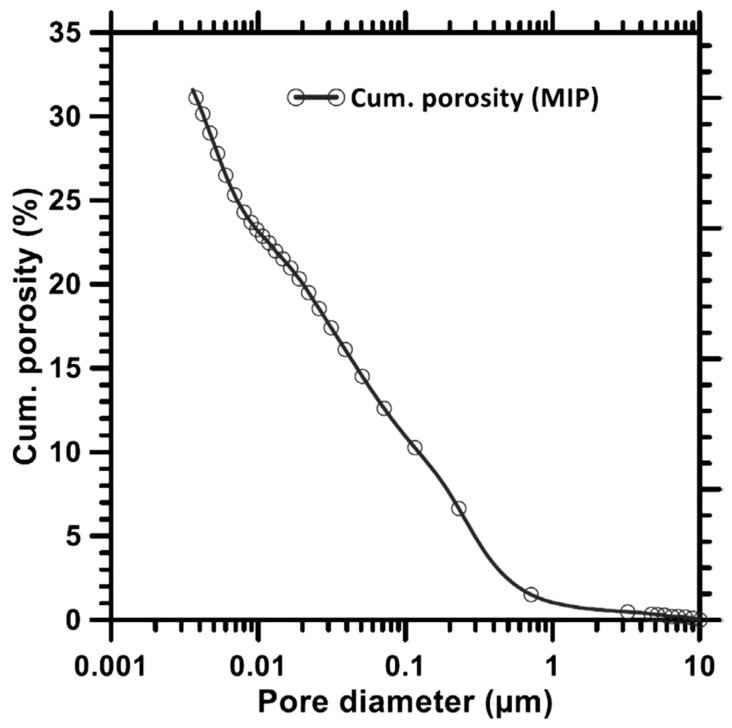
Cumulative mercury intrusion curve for alkali-activated FA. Adapted from [110].

The study by Aligizaki [263] showed that porosity can assist in understanding the characteristics of GP concrete and PC as it is a sign of the density of the Ms, microcrack presence, and diffusion rate of pore sol. Das, Yang, Singh, Mertens, Xiao, Chawla, and Neithalath [110] examined the dimension of the pore structure of FA-GP concrete in the range of 0.0036–10 µm MIP. As shown in Figure 26, the cumulative porosity for FA-GP concrete is 32%. However, the majority of the pores measured in the GP matrix were 0.0036–1 µm in size.

### 8.4. GP Concrete and PC Ms Comparison

**Figure 27:**
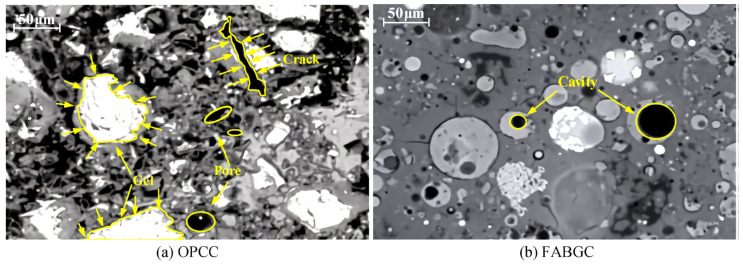
SEM images of the binders and ITZs of (**a**) PCC and (**b**) FA-GP concrete. Adapted from [172,259,265].

**Figure 28:**
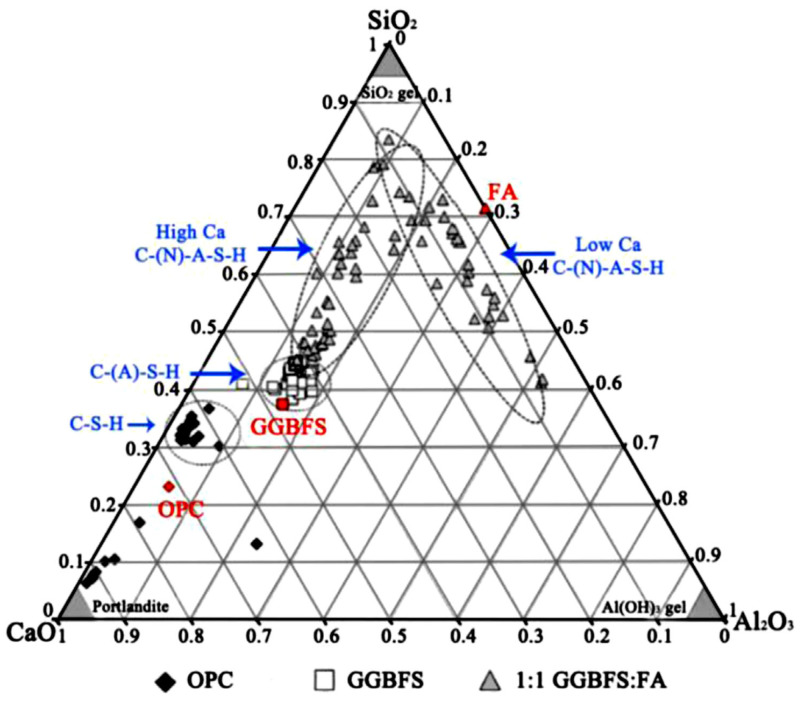
GP and PC binder gel compositions. Adapted from [66].

As demonstrated in Figure 27, hardened GP concrete has a denser Ms than hardened PC because of the previously noted cross-linked characteristic. Because of the replacement of Al^+3^ for Si^+4^ in bridging locations, the C-A-S-H matrix chains in the GP system were shown to be longer than the C-S-H gel chains in the PC system [35]. Because of the stronger interlayer cohesion caused by the bonding between bridging tetrahedral conservative layers, C-A-S-H gels have a lower Ca/Si ratio and a higher Al/Si ratio than C-S-H gels [264]. The gel composition of GP concrete and PC is shown in Figure 28.

## 9. Challenges for Solid Wastes in Geopolymer

To accomplish the industrialization and commercialization of geopolymers, hurdles must be overcome in three areas: technology, economy, and administration. This also necessitates collaboration between the government, businesses, and the general people for co-governance, which is both an objective necessity for enhancing the quality of the ecological environment and a practical prerequisite for constructing an ecological civilization. Geopolymers derived from solid wastes are expected to become more frequently employed when nonrenewable resources are depleted. Basic research should be prioritized in order to minimize the cost and increase the workability of geopolymers. Based on fundamental research, embarking on solid wastes to explore the preparation of varied characteristics of geopolymers in order to produce high-value-added application domains should be one of the primary paths of future geopolymer research development [1,266,267,268,269,270].

## 10. Conclusions

With the main alumino-silicate precursors used in the production of GP concrete being industrial wastes, such as FA, the utilization of GP concrete in place of the conventional PCC can be used to reduce CO_2_ emissions. The use of GP concrete would also result in an effective way to manage industrial wastes such as FA, thereby promoting the sustainable development of society. Discussions presented in this review paper showed that FA-GP concrete could be an alternative to PCC. Nevertheless, this will only happen if both an efficient raw material supply chain and a product supply chain network are in situ. From this perspective, the recent market is optimistic, but it will take time to establish GP concrete as a commercial product worldwide. Desirable performance for diverse engineering applications may be achieved with correct selection and adjustment of GP concrete composition. Regardless of the differences in the precursors’ characteristics, the production and formation and the characteristics of the FA-GP concrete products are highly influenced by the physical and chemical characteristics of FA, AAs, additives, and curing conditions. Based on the discussion in this paper, the following conclusions can be made:

1. The recommended S/A ratio for producing GPs of appropriate strength varies depending on the nature and content of the raw material. S/A ratios of 2–2.5 (class F FA) and 2.5–3.5 (class C FA) were determined to be optimal. The influence of Al and Si on setting and hardening characteristics in high-Ca FA is substantial.
2. The conventional AAs, a combination of NaOH and Na_2_SiO_3_, are very good and economically viable alkali activating sols. Microstructural investigations revealed that GP concrete prepared with a lower NaOH to Na_2_SiO_3_ ratio reacts more efficiently because of the large surface area available for reaction and binding without being clogged by excess water. However, the hydroxyl groups tend to condense when specimens are heated to high temperatures.
3. FA-ITZ GP concrete consists of gel, pores, fissures, and unreacted FA particles. N-A-S-H gel can improve the strength of the ITZ by promoting the combination of the aggregate interface and the GP matrix. As a result, FA-GP concrete has nearly no obvious weak ITZ near the aggregate.
4. The existence of C-S-H gel, as well as GP gel, improves the mechanical and Ms characteristics of precursors either having high Ca or mixed with Ca components.
5. When it comes to GP synthesis, the criticality of precursor oxide dosage cannot be overstated. In general, the Si oxide content should be between 45% and 55%, the Al dosage should be between 22% and 28%, and the FeCO_2_ and CaO content should be between 15% and 20%.
6. The experiment using MIP analysis reveals that extending the curing duration of GP specimens is useful in minimizing pores. The increased geopolymerization limits the connection between the pores, leading to a denser pattern. Furthermore, FA particles and gel may be used to refine the pores in FA-GP concrete. As a result, FA-GP concrete gel pores and capillary pores are concentrated in the tiny pore-size region. However, it is worth noting that the silicon content of the AA plays an essential role in the refining of the FA-GP concrete pore structure.
7. It was found that increasing the fineness leads to an increase in the reaction rate and therefore requires a minus time of heating to achieve strength, as it was found that more than 60 MPa can achieve the strength of GP concrete by increasing the fineness of the precursor material.
8. FA-GP concrete with a compact and denser structure shows high mechanical strength and good efflorescence that make it an ideal choice for the construction industry and has been effectively used in precast industries. Moreover, there is an enormous possibility of utilizing high-Ca GP as a restorative material.

## 11. Recommendations

Though FA-GP concrete is a relatively new, innovative, and sustainable engineered material combination with numerous advantages, some difficulties remain, and some are recommended below for further investigation:

1. Currently, the findings of relevant investigations on the Ms of FA-GP concrete are discordant in several respects. For example, the conclusions on forming a new crystalline phase prior to and during the AA reaction of FA are inconsistent. As a result, additional systematic and in-depth research is required to prove this substance’s usefulness with greater certainty. Additionally, advanced analytical techniques such as micro- or nano-analysis and nuclear magnetic resonance must be employed.
2. Suitable guidelines for the selection of aggregate contents in FA-GP concrete should be developed. It is also critical to develop design procedures for each substantial predecessor. Furthermore, the usage of dune sands, fibers, marble dust, date ash, and other materials as precursors should be investigated.
3. Detailed investigations, such as those relating to the derivation and modeling of reaction kinetics under different treatment and production circumstances of the developing class of GP raw material, such as blended GPs and biomass ash GPs, are needed.
4. GP binders need a high pH and heat curing. As a result, efforts are needed to create a room-temperature-cured one-component GP system that uses solid activators rather than alkaline sols in order to gain widespread acceptability in the field.
5. FA-GP concrete should be given more functionality instead of using it as an alternative cement only. FA-GP concrete with biomass has the potential for development as a new class of lightweight fireproof composites.
6. Lastly, more research is needed to evaluate the cost of GP concrete compared to that of standard concrete. Furthermore, contradictory findings regarding the embodied energy and carbon footprint of GPs in comparison to traditional Portland cement must be addressed.

## Figures and Tables

**Table 1:** FA classification and applications for the production of inorganic GPs. Adapted from [91].

FAs	Origin	Characteristic	Mineral Phases	Potential of Products	Potential for Geopolymerization
1. Pozzolanic (sialic)	Highly ranked fuels, bituminous with detrital quartz, kaolinite, illite, mica, k-feldspars	Maximum values of fine fraction, true density	Viscous melt (glass), minimum values of crystallization	Dense and porous GP pastes	Good activation but with a low rate of high dissolution pH of alkaline sol
2. Active low pozzolanic, calsialic, ferrisialic, ferricalsialic	Variable rank of fuel but mostly liquid with carbonates, sulfates, sulfides, detrital silicates	Maximum values of the water-soluble fraction	Oxyhydrosydes, sulfates, carb, and actives silicates	Porous matrix paste	Aam low alkaline sol and pH
3. Ferrisialic inert (sialic), calsialic	Variable rank of fuels, bituminous and sub-bituminous	High content of original infused quartz, max true density, min of water-soluble elements	Mullite and quartz	Dense and less porous composites	Low dissolution rate, high pH
4. Ferricalsialic	Low-rank fuels, lignites, bituminous gypsum, ferrite, calcite, dolomite	Max bulk density	Fe, Mg, Na phases	Porous product paste	High dissolution rate, poor polymerization
5. Sialic and calsialic mixed between inert and active	Lower-rank fuel, lignites and sub-bituminous with carbonate and sulfates	Maximum values of light fraction	Oxyhydroxides sulfates, carbonates, and active Ca and Caemg silicates	Paste and mortars, porous metallic corrosion, and pores	A medium reactive low alkaline sol

## Data Availability

The data used to support the findings of this study are included in the article.

## References

[1] Ahmed H.U., Mohammed A.S., Qaidi S.M.A., Faraj R.H., Sor N.H., Mohammed A.A. (2022). Compressive strength of geopolymer concrete composites: A systematic comprehensive review, analysis and modeling. Eur. J. Environ. Civ. Eng..

[2] Ahmad J., Kontoleon K.J., Majdi A., Naqash M.T., Deifalla A.F., Ben Kahla N., Isleem H.F., Qaidi S.M.A. (2022). A Comprehensive Review on the Ground Granulated Blast Furnace Slag (GGBS) in Concrete Production. Sustainability.

[3] Saeed A., Najm H.M., Hassan A., Qaidi S., Sabri M.M.S., Mashaan N.S. (2022). A Comprehensive Study on the Effect of Regular and Staggered Openings on the Seismic Performance of Shear Walls. Buildings.

[4] Elsheikh A. (2022). Bistable Morphing Composites for Energy-Harvesting Applications. Polymers.

[5] Qaidi S. (2021). Behaviour of Concrete Made of Recycled Waste PET and Confined with CFRP Fabrics. Master’s Thesis.

[6] Monteiro P.J.M., Miller S.A., Horvath A. (2017). Towards sustainable concrete. Nat. Mater..

[7] Arshad S., Sharif M.B., Irfan-Ul-Hassan M., Khan M., Zhang J.-L. (2020). Efficiency of Supplementary Cementitious Materials and Natural Fiber on Mechanical Performance of Concrete. Arab. J. Sci. Eng..

[8] Cao M., Khan M. (2021). Effectiveness of multiscale hybrid fiber reinforced cementitious composites under single degree of freedom hydraulic shaking table. Struct. Concr..

[9] Cao M., Khan M., Ahmed S. (2020). Effectiveness of Calcium Carbonate Whisker in Cementitious Composites. Period. Polytech. Civ. Eng..

[10] A Miller S., Horvath A., Monteiro P.J.M. (2016). Readily implementable techniques can cut annual CO_2_ emissions from the production of concrete by over 20%. Environ. Res. Lett..

[11] Miller S.A., Moore F.C. (2020). Climate and health damages from global concrete production. Nat. Clim. Chang..

[12] Ahmad J., Aslam F., Martinez-Garcia R., De-Prado-Gil J., Qaidi S.M.A., Brahmia A. (2021). Effects of waste glass and waste marble on mechanical and durability performance of concrete. Sci. Rep..

[13] Gollakota A.R., Volli V., Shu C.-M. (2019). Progressive utilisation prospects of coal fly ash: A review. Sci. Total Environ..

[14] Amran Y.M., Alyousef R., Alabduljabbar H., El-Zeadani M. (2020). Clean production and properties of geopolymer concrete; A review. J. Clean. Prod..

[15] Xiao R., Polaczyk P., Zhang M., Jiang X., Zhang Y., Huang B., Hu W. (2020). Evaluation of Glass Powder-Based Geopolymer Stabilized Road Bases Containing Recycled Waste Glass Aggregate. Transp. Res. Rec. J. Transp. Res. Board.

[16] Ng C., Alengaram U.J., Wong L.S., Mo K.H., Jumaat M.Z., Ramesh S. (2018). A review on microstructural study and compressive strength of geopolymer mortar, paste and concrete. Constr. Build. Mater..

[17] Albidah A.S. (2021). Effect of partial replacement of geopolymer binder materials on the fresh and mechanical properties: A review. Ceram. Int..

[18] Raj M.K.A., Muthusamy S., Panchal H., Ibrahim A.M.M., Alsoufi M.S., Elsheikh A.H. (2022). Investigation of mechanical properties of dual-fiber reinforcement in polymer composite. J. Mater. Res. Technol..

[19] Kamal A., Ashmawy M., Shanmugan S., Algazzar A.M., Elsheikh A.H. (2021). Fabrication techniques of polymeric nanocomposites: A comprehensive review. Proc. Inst. Mech. Eng. Part C J. Mech. Eng. Sci..

[20] Elsheikh A.H., Panchal H., Shanmugan S., Muthuramalingam T., El-Kassas A., Ramesh B. (2022). Recent progresses in wood-plastic composites: Pre-processing treatments, manufacturing techniques, recyclability and eco-friendly assessment. Clean. Eng. Technol..

[21] Assi L., Carter K., Deaver E., Anay R., Ziehl P. (2018). Sustainable concrete: Building a greener future. J. Clean. Prod..

[22] Assi L.N., Carter K., Deaver E., Ziehl P. (2020). Review of availability of source materials for geopolymer/sustainable concrete. J. Clean. Prod..

[23] Van Jaarsveld J.G.S., van Deventer J.S.J., Lukey G.C. (2003). The characterisation of source materials in fly ash-based geopolymers. Mater. Lett..

[24] Ahmad J., Majdi A., Elhag A.B., Deifalla A.F., Soomro M., Isleem H.F., Qaidi S. (2022). A Step towards Sustainable Concrete with Substitution of Plastic Waste in Concrete: Overview on Mechanical, Durability and Microstructure Analysis. Crystals.

[25] Ahmed H.U., Mohammed A.A., Rafiq S., Mohammed A.S., Mosavi A., Sor N.H., Qaidi S.M.A. (2021). Compressive Strength of Sustainable Geopolymer Concrete Composites: A State-of-the-Art Review. Sustainability.

[26] Ahmed H.U., Mohammed A.S., Faraj R.H., Qaidi S.M., Mohammed A.A. (2022). Compressive strength of geopolymer concrete modified with nano-silica: Experimental and modeling investigations. Case Stud. Constr. Mater..

[27] Davidovits J. (1991). Geopolymers. J. Therm. Anal..

[28] Barbosa V.F., MacKenzie K. (2003). Thermal behaviour of inorganic geopolymers and composites derived from sodium polysialate. Mater. Res. Bull..

[29] Phair J., Van Deventer J. (2001). Effect of silicate activator pH on the leaching and material characteristics of waste-based inorganic polymers. Miner. Eng..

[30] Najm H.M., Nanayakkara O., Sabri M.M.S. (2022). Destructive and Non-Destructive Evaluation of Fibre-Reinforced Concrete: A Comprehensive Study of Mechanical Properties. Materials.

[31] Khan M., Ali M. (2019). Improvement in concrete behavior with fly ash, silica-fume and coconut fibres. Constr. Build. Mater..

[32] Khan M., Ali M. (2020). Optimization of concrete stiffeners for confined brick masonry structures. J. Build. Eng..

[33] Rodgers L. (2018). Climate change: The massive CO_2_ emitter you may not know about. BBC News.

[34] Farhan K.Z., Johari M.A.M., Demirboğa R. (2020). Assessment of important parameters involved in the synthesis of geopolymer composites: A review. Constr. Build. Mater..

[35] Hassan A., Arif M., Shariq M. (2019). Use of geopolymer concrete for a cleaner and sustainable environment—A review of mechanical properties and microstructure. J. Clean. Prod..

[36] Mohajerani A., Suter D., Jeffrey-Bailey T., Song T., Arulrajah A., Horpibulsuk S., Law D. (2019). Recycling waste materials in geopolymer concrete. Clean Technol. Environ. Policy.

[37] Ahmed S.N., Sor N.H., Ahmed M.A., Qaidi S.M. (2022). Thermal conductivity and hardened behavior of eco-friendly concrete incorporating waste polypropylene as fine aggregate. Mater. Today Proc..

[38] Aslam F., Zaid O., Althoey F., Alyami S.H., Qaidi S.M.A., de Prado Gil J., Martínez-García R. (2022). Evaluating the influence of fly ash and waste glass on the characteristics of coconut fibers reinforced concrete. Struct. Concrete.

[39] Ryu G.S., Lee Y.B., Koh K.T., Chung Y.S. (2013). The mechanical properties of fly ash-based geopolymer concrete with alkaline activators. Constr. Build. Mater..

[40] Sreevidya V. (2014). Investigations on the Flexural Behaviour of Ferro Geopolymer Composite Slabs.

[41] Emad W., Mohammed A.S., Bras A., Asteris P.G., Kurda R., Muhammed Z., Hassan A., Qaidi S.M., Sihag P. (2022). Metamodel techniques to estimate the compressive strength of UHPFRC using various mix proportions and a high range of curing temperatures. Constr. Build. Mater..

[42] Faraj R.H., Ahmed H.U., Rafiq S., Sor N.H., Ibrahim D.F., Qaidi S.M. (2022). Performance of Self-Compacting mortars modified with Nanoparticles: A systematic review and modeling. Clean. Mater..

[43] He X., Yuhua Z., Qaidi S., Isleem H.F., Zaid O., Althoey F., Ahmad J. (2022). Mine tailings-based geopolymers: A comprehensive review. Ceram. Int..

[44] Maglad A.M., Zaid O., Arbili M.M., Ascensão G., Șerbănoiu A.A., Grădinaru C.M., García R.M., Qaidi S.M.A., Althoey F., de Prado-Gil J. (2022). A Study on the Properties of Geopolymer Concrete Modified with Nano Graphene Oxide. Buildings.

[45] Mansi A., Sor N.H., Hilal N., A Qaidi S.M. The Impact of Nano Clay on Normal and High-Performance Concrete Characteristics: A Review. Proceedings of the 2nd International Conference of Al-Esraa University College for Engineering Sciences (ICAUC_ES 2021).

[46] Martínez-García R., Jagadesh P., Zaid O., Șerbănoiu A.A., Fraile-Fernández F.J., de Prado-Gil J., Qaidi S.M.A., Grădinaru C.M. (2022). The Present State of the Use of Waste Wood Ash as an Eco-Efficient Construction Material: A Review. Materials.

[47] Qaidi S. (2022). Ultra-high-performance fiber-reinforced concrete (UHPFRC): A mini-review of the challenges. ScienceOpen Preprints.

[48] Qaidi S. (2022). Ultra-high-performance fiber-reinforced concrete: Applications. Preprints.

[49] Hemalatha T., Ramaswamy A. (2017). A review on fly ash characteristics—Towards promoting high volume utilization in developing sustainable concrete. J. Clean. Prod..

[50] Dwivedi A., Jain M.K. (2014). Fly ash–waste management and overview: A Review. Recent Res. Sci. Technol..

[51] Amran M., Debbarma S., Ozbakkaloglu T. (2021). Fly ash-based eco-friendly geopolymer concrete: A critical review of the long-term durability properties. Constr. Build. Mater..

[52] Ranjbar N., Kuenzel C. (2017). Cenospheres: A review. Fuel.

[53] Arnett D.K., Blumenthal R.S., Albert M.A., Buroker A.B., Goldberger Z.D., Hahn E.J., Himmelfarb C.D., Khera A., Lloyd-Jones D., McEvoy J.W. (2019). 2019 ACC/AHA Guideline on the Primary Prevention of Cardiovascular Disease: A Report of the American College of Cardiology/American Heart Association Task Force on Clinical Practice Guidelines. Circulation.

[54] Nanayakkara O., Najm H.M., Sabri M.M.S. (2022). Effect of Using Steel Bar Reinforcement on Concrete Quality by Ultrasonic Pulse Velocity Measurements. Materials.

[55] Qaidi S.M.A. (2021). PET-Concrete.

[56] Davidovits J. (1989). Geopolymers and geopolymeric materials. J. Therm. Anal..

[57] Purdon A. (1940). The action of alkalis on blast-furnace slag. J. Soc. Chem. Ind..

[58] Shi C., Roy D., Krivenko P. (2003). Alkali-Activated Cements and Concretes.

[59] Provis J.L., Van Deventer J.S. (2013). Alkali Activated Materials: State-of-the-Art Report.

[60] Pacheco-Torgal E., Labrincha J., Leonelli C., Palomo A., Chindaprasit P. (2014). Handbook of Alkali-Activated Cements, Mortars and Concretes.

[61] Khan M., Cao M., Ai H., Hussain A. (2022). Basalt Fibers in Modified Whisker Reinforced Cementitious Composites. Period. Polytech. Civ. Eng..

[62] Khan M., Cao M., Ali M. (2018). Experimental and Empirical Study of Basalt Fibber Reinforced Concrete.

[63] Khan M., Cao M., Chu S., Ali M. (2022). Properties of hybrid steel-basalt fiber reinforced concrete exposed to different surrounding conditions. Constr. Build. Mater..

[64] Provis J.L., van Deventer J.S.J. (2007). Geopolymerisation kinetics. 2. Reaction kinetic modelling. Chem. Eng. Sci..

[65] Provis J.L., van Deventer J.S.J. (2007). Geopolymerisation kinetics. 1. In situ energy-dispersive X-ray diffractometry. Chem. Eng. Sci..

[66] Zhang P., Gao Z., Wang J., Guo J., Hu S., Ling Y. (2020). Properties of fresh and hardened fly ash/slag based geopolymer concrete: A review. J. Clean. Prod..

[67] Qaidi S.M.A. (2021). PET-Concrete Confinement with CFRP.

[68] Qaidi S.M.A. (2022). Ultra-High-Performance Fiber-Reinforced Concrete: Principles and Raw Materials.

[69] Singh J., Singh S.P. (2019). Geopolymerization of solid waste of non-ferrous metallurgy—A review. J. Environ. Manag..

[70] Shi C., Jimenez A.F., Palomo A. (2011). New cements for the 21st century: The pursuit of an alternative to Portland cement. Cem. Concr. Res..

[71] Liu M.Y.J., Alengaram U.J., Santhanam M., Jumaat M.Z., Mo K.H. (2016). Microstructural investigations of palm oil fuel ash and fly ash based binders in lightweight aggregate foamed geopolymer concrete. Constr. Build. Mater..

[72] Zhang Y., Xiao R., Jiang X., Li W., Zhu X., Huang B. (2020). Effect of particle size and curing temperature on mechanical and microstructural properties of waste glass-slag-based and waste glass-fly ash-based geopolymers. J. Clean. Prod..

[73] Gupta V., Pathak D.K., Siddique S., Kumar R., Chaudhary S. (2019). Study on the mineral phase characteristics of various Indian biomass and coal fly ash for its use in masonry construction products. Constr. Build. Mater..

[74] Hashmi A.F., Shariq M., Baqi A., Haq M. (2020). Optimization of fly ash concrete mix—A solution for sustainable development. Mater. Today Proc..

[75] Khan M., Cao M., Hussain A., Chu S. (2021). Effect of silica-fume content on performance of CaCO_3_ whisker and basalt fiber at matrix interface in cement-based composites. Constr. Build. Mater..

[76] Khan M., Cao M., Xie C., Ali M. (2022). Effectiveness of hybrid steel-basalt fiber reinforced concrete under compression. Case Stud. Constr. Mater..

[77] Khan M., Cao M., Xie C., Ali M. (2021). Hybrid fiber concrete with different basalt fiber length and content. Struct. Concr..

[78] Yin K., Ahamed A., Lisak G. (2018). Environmental perspectives of recycling various combustion ashes in cement production—A review. Waste Manag..

[79] Wang T., Ishida T., Gu R. (2020). A study of the influence of crystal component on the reactivity of low-calcium fly ash in alkaline conditions based on SEM-EDS. Constr. Build. Mater..

[80] Yilmaz G. (2012). Structural characterization of glass–ceramics made from fly ash containing SiO_2_–Al_2_O_3_–Fe_2_O_3_–CaO and analysis by FT-IR–XRD–SEM methods. J. Mol. Struct..

[81] Qaidi S.M.A. (2022). Ultra-High-Performance Fiber-Reinforced Concrete: Mixture Design.

[82] Qaidi S.M.A. (2022). Ultra-High-Performance Fiber-Reinforced Concrete: Hydration and Microstructure.

[83] Qaidi S.M.A. (2022). Ultra-High-Performance Fiber-Reinforced Concrete: Fresh Properties.

[84] Chou M.I.M. (2012). Fly Ash, Encyclopedia of Sustainability Scienc and Technology.

[85] Davidovits J. (2002). 30 years of successes and failures in geopolymer applications. Market trends and potential breakthroughs. Geopolymer 2002 Conference.

[86] Dimas D., Giannopoulou I., Panias D. (2009). Polymerization in sodium silicate solutions: A fundamental process in geopolymerization technology. J. Mater. Sci..

[87] He J., Zhang J., Yu Y., Zhang G. (2012). The strength and microstructure of two geopolymers derived from metakaolin and red mud-fly ash admixture: A comparative study. Constr. Build. Mater..

[88] Kriven W.M., Bell J.L., Gordon M. (2003). Microstructure and Microchemistry of Fully-Reacted Geopolymers and Geopolymer Matrix Composites. Ceram. Trans..

[89] Duxson P., Provis J.L., Lukey G.C., Mallicoat S.W., Kriven W.M., van Deventer J.S.J. (2005). Understanding the relationship between geopolymer composition, microstructure and mechanical properties. Colloids Surf. A Physicochem. Eng. Asp..

[90] Zhuang X.Y., Chen L., Komarneni S., Zhou C.H., Tong D.S., Yang H.M., Yu W.H., Wang H. (2016). Fly ash-based geopolymer: Clean production, properties and applications. J. Clean. Prod..

[91] Kamseu E., Alzari V., Nuvoli D., Sanna D., Lancellotti I., Mariani A., Leonelli C. (2021). Dependence of the geopolymerization process and end-products to the nature of solid precursors: Challenge of the sustainability. J. Clean. Prod..

[92] Ren B., Zhao Y., Bai H., Kang S., Zhang T., Song S. (2021). Eco-friendly geopolymer prepared from solid wastes: A critical review. Chemosphere.

[93] Okoye F., Durgaprasad J., Singh N. (2016). Effect of silica fume on the mechanical properties of fly ash based-geopolymer concrete. Ceram. Int..

[94] Kong D.L., Sanjayan J.G. (2010). Effect of elevated temperatures on geopolymer paste, mortar and concrete. Cem. Concr. Res..

[95] Pavithra P., Reddy M.S., Dinakar P., Rao B.H., Satpathy B., Mohanty A. (2016). A mix design procedure for geopolymer concrete with fly ash. J. Clean. Prod..

[96] Xie T., Ozbakkaloglu T. (2015). Behavior of low-calcium fly and bottom ash-based geopolymer concrete cured at ambient temperature. Ceram. Int..

[97] Abdulkareem O.A., Al Bakri A.M., Kamarudin H., Nizar I.K., Saif A.A. (2014). Effects of elevated temperatures on the thermal behavior and mechanical performance of fly ash geopolymer paste, mortar and lightweight concrete. Constr. Build. Mater..

[98] Assi L., Ghahari S., Deaver E., Leaphart D., Ziehl P. (2016). Improvement of the early and final compressive strength of fly ash-based geopolymer concrete at ambient conditions. Constr. Build. Mater..

[99] Adak D., Sarkar M., Mandal S. (2017). Structural performance of nano-silica modified fly-ash based geopolymer concrete. Constr. Build. Mater..

[100] Embong R., Kusbiantoro A., Shafiq N., Nuruddin M.F. (2016). Strength and microstructural properties of fly ash based geopolymer concrete containing high-calcium and water-absorptive aggregate. J. Clean. Prod..

[101] Ranjbar N., Mehrali M., Alengaram U.J., Metselaar H.S.C., Jumaat M.Z. (2014). Compressive strength and microstructural analysis of fly ash/palm oil fuel ash based geopolymer mortar under elevated temperatures. Constr. Build. Mater..

[102] Duan P., Yan C., Luo W., Zhou W. (2016). Effects of adding nano-TiO_2_ on compressive strength, drying shrinkage, carbonation and microstructure of fluidized bed fly ash based geopolymer paste. Constr. Build. Mater..

[103] Park Y., Abolmaali A., Kim Y.H., Ghahremannejad M. (2016). Compressive strength of fly ash-based geopolymer concrete with crumb rubber partially replacing sand. Constr. Build. Mater..

[104] Assaedi H., Shaikh F.U.A., Low I.M. (2016). Influence of mixing methods of nano silica on the microstructural and mechanical properties of flax fabric reinforced geopolymer composites. Constr. Build. Mater..

[105] Khan M., Castel A., Akbarnezhad A., Foster S., Smith M. (2016). Utilisation of steel furnace slag coarse aggregate in a low calcium fly ash geopolymer concrete. Cem. Concr. Res..

[106] Leong H.Y., Ong D.E.L., Sanjayan J., Nazari A. (2016). Suitability of Sarawak and Gladstone fly ash to produce geopolymers: A physical, chemical, mechanical, mineralogical and microstructural analysis. Ceram. Int..

[107] Gunasekara C., Law D., Setunge S. (2016). Long term permeation properties of different fly ash geopolymer concretes. Constr. Build. Mater..

[108] Zeng S., Wang J. (2016). Characterization of mechanical and electric properties of geopolymers synthesized using four locally available fly ashes. Constr. Build. Mater..

[109] Huiskes D.M.A., Keulen A., Yu Q.L., Brouwers H.J.H. (2016). Design and performance evaluation of ultra-lightweight geopolymer concrete. Mater. Des..

[110] Das S., Yang P., Singh S.S., Mertens J.C., Xiao X., Chawla N., Neithalath N. (2015). Effective properties of a fly ash geopolymer: Synergistic application of X-ray synchrotron tomography, nanoindentation, and homogenization models. Cem. Concr. Res..

[111] Tho-In T., Sata V., Boonserm K., Chindaprasirt P. (2018). Compressive strength and microstructure analysis of geopolymer paste using waste glass powder and fly ash. J. Clean. Prod..

[112] Pilehvar S., Cao V.D., Szczotok A.M., Carmona M., Valentini L., Lanzón M., Pamies R., Kjøniksen A.-L. (2018). Physical and mechanical properties of fly ash and slag geopolymer concrete containing different types of micro-encapsulated phase change materials. Constr. Build. Mater..

[113] Jindal B.B. (2018). Feasibility study of ambient cured geopolymer concrete—A review. Adv. Concr. Constr..

[114] Hussein O.H., Ibrahim A.M., Abd S.M., Najm H.M., Shamim S., Sabri M.M.S. (2022). Hybrid Effect of Steel Bars and PAN Textile Reinforcement on Ductility of One-Way Slab Subjected to Bending. Molecules.

[115] Najm H.M., Ahmad S. (2021). The effect of metallic and non-metallic fiber on the mechanical properties of waste ceramic concrete. Innov. Infrastruct. Solut..

[116] Qaidi S., Najm H.M., Abed S.M., Özkılıç Y.O., Al Dughaishi H., Alosta M., Sabri M.M.S., Alkhatib F., Milad A. (2022). Concrete Containing Waste Glass as an Environmentally Friendly Aggregate: A Review on Fresh and Mechanical Characteristics. Materials.

[117] Pimraksa K., Chindaprasirt P., Rungchet A., Sagoe-Crentsil K., Sato T. (2011). Lightweight geopolymer made of highly porous siliceous materials with various Na_2_O/Al_2_O_3_ and SiO_2_/Al_2_O_3_ ratios. Mater. Sci. Eng. A.

[118] Khan M., Lao J., Dai J.-G. (2022). Comparative study of advanced computational techniques for estimating the compressive strength of UHPC. J. Asian Concr. Fed..

[119] Khan M., Rehman A., Ali M. (2020). Efficiency of silica-fume content in plain and natural fiber reinforced concrete for concrete road. Constr. Build. Mater..

[120] Khan U.A., Jahanzaib H.M., Khan M., Ali M. (2018). Improving the Tensile Energy Absorption of High Strength Natural Fiber Reinforced Concrete with Fly-Ash for Bridge Girders. Key Engineering Materials.

[121] Parvez I., Shen J., Khan M., Cheng C. (2019). Modeling and Solution Techniques Used for Hydro Generation Scheduling. Water.

[122] Al Bakri A.M., Kamarudin H., Bnhussain M., Nizar I.K., Rafiza A., Zarina Y. (2012). The processing, characterization, and properties of fly ash based geopolymer concrete. Rev. Adv. Mater. Sci..

[123] Songpiriyakij S., Kubprasit T., Jaturapitakkul C., Chindaprasirt P. (2010). Compressive strength and degree of reaction of biomass- and fly ash-based geopolymer. Constr. Build. Mater..

[124] Hardjito D., Rangan B.V. (2005). Development and Properties of Low-Calcium Fly Ash-Based Geopolymer Concrete.

[125] Olivia M., Nikraz H. (2012). Properties of fly ash geopolymer concrete designed by Taguchi method. Mater. Des..

[126] Luhar I., Luhar S. (2022). A Comprehensive Review on Fly Ash-Based Geopolymer. J. Compos. Sci..

[127] Wallah S., Rangan B.V. (2006). Low-Calcium Fly Ash-Based Geopolymer Concrete: Long-Term Properties.

[128] Lee N., Jang J., Lee H. (2014). Shrinkage characteristics of alkali-activated fly ash/slag paste and mortar at early ages. Cem. Concr. Compos..

[129] Kuenzel C., Neville T., Donatello S., Vandeperre L., Boccaccini A., Cheeseman C. (2013). Influence of metakaolin characteristics on the mechanical properties of geopolymers. Appl. Clay Sci..

[130] Zhang Z., Zhu H., Zhou C., Wang H. (2016). Geopolymer from kaolin in China: An overview. Appl. Clay Sci..

[131] Joseph B., Mathew G. (2015). Behavior of Geopolymer Concrete Exposed to Elevated Temperatures. Ph.D Thesis.

[132] Ramujee K., PothaRaju M. (2017). Mechanical Properties of Geopolymer Concrete Composites. Mater. Today Proc..

[133] Collins F., Sanjayan J. (1998). Early Age Strength and Workability of Slag Pastes Activated by NaOH and Na_2_CO_3_. Cem. Concr. Res..

[134] June J. Geopolymer Concrete with Fly Ash. Proceedings of the Second International Conference on Sustainable Construction Materials and Technologies.

[135] Ferdous W., Manalo A., Khennane A., Kayali O. (2015). Geopolymer concrete-filled pultruded composite beams—Concrete mix design and application. Cem. Concr. Compos..

[136] Anuradha R., Sreevidya V., Venkatasubramani R., Rangan B.V. (2012). Modified guidelines for geopolymer concrete mix design using Indian standard. Asian J. Civ. Eng..

[137] Lokuge W., Wilson A., Gunasekara C., Law D.W., Setunge S. (2018). Design of fly ash geopolymer concrete mix proportions using Multivariate Adaptive Regression Spline model. Constr. Build. Mater..

[138] Mehta A., Siddique R., Singh B.P., Aggoun S., Łagód G., Barnat-Hunek D. (2017). Influence of various parameters on strength and absorption properties of fly ash based geopolymer concrete designed by Taguchi method. Constr. Build. Mater..

[139] Davidovits J. (2008). Geopolymer Chemistry and Applications. Geopolymer Institute.

[140] Phoo-Ngernkham T., Phiangphimai C., Damrongwiriyanupap N., Hanjitsuwan S., Thumrongvut J., Chindaprasirt P. (2018). A Mix Design Procedure for Alkali-Activated High-Calcium Fly Ash Concrete Cured at Ambient Temperature. Adv. Mater. Sci. Eng..

[141] Li N., Shi C., Zhang Z., Wang H., Liu Y. (2019). A review on mixture design methods for geopolymer concrete. Compos. Part B Eng..

[142] Nuaklong P., Sata V., Chindaprasirt P. (2016). Influence of recycled aggregate on fly ash geopolymer concrete properties. J. Clean. Prod..

[143] Nath P., Sarker P.K. (2014). Effect of GGBFS on setting, workability and early strength properties of fly ash geopolymer concrete cured in ambient condition. Constr. Build. Mater..

[144] Noushini A., Castel A., Aldred J., Rawal A. (2020). Chloride diffusion resistance and chloride binding capacity of fly ash-based geopolymer concrete. Cem. Concr. Compos..

[145] Farhan N.A., Sheikh M.N., Hadi M.N. (2018). Experimental Investigation on the Effect of Corrosion on the Bond Between Reinforcing Steel Bars and Fibre Reinforced Geopolymer Concrete. Structures.

[146] Al-Azzawi M., Yu T., Hadi M.N. (2018). Factors Affecting the Bond Strength Between the Fly Ash-based Geopolymer Concrete and Steel Reinforcement. Structures.

[147] Okoye F.N., Prakash S., Singh N.B. (2017). Durability of fly ash based geopolymer concrete in the presence of silica fume. J. Clean. Prod..

[148] Memon F., Nuruddin F., Shafiq N. (2011). Compressive strength and workability characteristics of low-calcium fly ash-based self-compacting geopolymer concrete. Int. J. Civ. Environ. Eng..

[149] Joseph B., Mathew G. (2012). Influence of aggregate content on the behavior of fly ash based geopolymer concrete. Sci. Iran..

[150] Kusbiantoro A., Nuruddin M.F., Shafiq N., Qazi S.A. (2012). The effect of microwave incinerated rice husk ash on the compressive and bond strength of fly ash based geopolymer concrete. Constr. Build. Mater..

[151] Nath P., Sarker P.K. (2015). Use of OPC to improve setting and early strength properties of low calcium fly ash geopolymer concrete cured at room temperature. Cem. Concr. Compos..

[152] Nuruddin M.F., Qazi S.A., Kusbiantoro A., Shafiq N. (2011). Utilisation of waste material in geopolymeric concrete. Proc. Inst. Civ. Eng. Constr. Mater..

[153] Rahman M., Sarker P. Geopolymer concrete columns under combined axial load and biaxial bending. Proceedings of the CONCRETE 2011 Conference.

[154] Rangan B. (2014). Geopolymer concrete for environmental protection. Indian Concr. J..

[155] Görhan G., Kürklü G. (2014). The influence of the NaOH solution on the properties of the fly ash-based geopolymer mortar cured at different temperatures. Compos. Part B Eng..

[156] Ahmari S., Zhang L. (2012). Production of eco-friendly bricks from copper mine tailings through geopolymerization. Constr. Build. Mater..

[157] Somna K., Jaturapitakkul C., Kajitvichyanukul P., Chindaprasirt P. (2011). NaOH-activated ground fly ash geopolymer cured at ambient temperature. Fuel.

[158] Sathonsaowaphak A., Chindaprasirt P., Pimraksa K. (2009). Workability and strength of lignite bottom ash geopolymer mortar. J. Hazard. Mater..

[159] Anuar K., Ridzuan A., Ismail S. (2011). Strength characteristic of geopolymer concrete containing recycled concrete aggregate. Int. J. Civ. Environ. Eng..

[160] Posi P., Teerachanwit C., Tanutong C., Limkamoltip S., Lertnimoolchai S., Sata V., Chindaprasirt P. (2013). Lightweight geopolymer concrete containing aggregate from recycle lightweight block. Mater. Des..

[161] Sata V., Wongsa A., Chindaprasirt P. (2013). Properties of pervious geopolymer concrete using recycled aggregates. Constr. Build. Mater..

[162] Shi X.S., Wang Q.Y., Zhao X.L., Collins F. (2012). Discussion on Properties and Microstructure of Geopolymer Concrete Containing Fly Ash and Recycled Aggregate. Adv. Mater. Res..

[163] Qaidi S.M.A. (2022). Ultra-High-Performance Fiber-Reinforced Concrete: Applications.

[164] Qaidi S.M., Atrushi D.S., Mohammed A.S., Ahmed H.U., Faraj R.H., Emad W., Najm H.M. (2022). Ultra-high-performance geopolymer concrete: A review. Constr. Build. Mater..

[165] Ahmed H.U., Mahmood L.J., Muhammad M.A., Faraj R.H., Qaidi S.M., Sor N.H., Mohammed A.S., Mohammed A.A. (2022). Geopolymer concrete as a cleaner construction material: An overview on materials and structural performances. Clean. Mater..

[166] De Vargas A.S., Dal Molin D.C.C., Vilela A.C.F., Silva F.J.D., Pavão B., Veit H. (2011). The effects of Na2O/SiO_2_ molar ratio, curing temperature and age on compressive strength, morphology and microstructure of alkali-activated fly ash-based geopolymers. Cem. Concr. Compos..

[167] Criado M., Palomo A., Fernández-Jiménez A. (2005). Alkali activation of fly ashes. Part 1: Effect of curing conditions on the carbonation of the reaction products. Fuel.

[168] Rattanasak U., Chindaprasirt P. (2009). Influence of NaOH solution on the synthesis of fly ash geopolymer. Miner. Eng..

[169] De Silva P., Sagoe-Crenstil K., Sirivivatnanon V. (2007). Kinetics of geopolymerization: Role of Al_2_O_3_ and SiO_2_. Cem. Concr. Res..

[170] North M.R., Swaddle T.W. (2000). Kinetics of Silicate Exchange in Alkaline Aluminosilicate Solutions. Inorg. Chem..

[171] Lizcano M., Kim H.S., Basu S., Radovic M. (2012). Mechanical properties of sodium and potassium activated metakaolin-based geopolymers. J. Mater. Sci..

[172] Ma Y., Hu J., Ye G. (2013). The pore structure and permeability of alkali activated fly ash. Fuel.

[173] Qaidi S. (2022). Ultra-High-Performance Geopolymer Concrete. Part 1: Manufacture Approaches.

[174] Qaidi S. (2022). Ultra-High-Performance Geopolymer Concrete. Part 2: Applications.

[175] Mijarsh M., Johari M.M., Ahmad Z.A. (2014). Synthesis of geopolymer from large amounts of treated palm oil fuel ash: Application of the Taguchi method in investigating the main parameters affecting compressive strength. Constr. Build. Mater..

[176] Phoo-ngernkham T., Chindaprasirt P., Sata V., Hanjitsuwan S., Hatanaka S. (2014). The effect of adding nano-SiO_2_ and nano-Al_2_O_3_ on properties of high calcium fly ash geopolymer cured at ambient temperature. Mater. Des..

[177] Rattanasak U., Chindaprasirt P., Suwanvitaya P. (2010). Development of high volume rice husk ash alumino silicate composites. Int. J. Miner. Met. Mater..

[178] Zhang Y.J., Wang Y.C., Xu D.L., Li S. (2010). Mechanical performance and hydration mechanism of geopolymer composite reinforced by resin. Mater. Sci. Eng. A.

[179] MacKenzie K.J., Komphanchai S., Vagana R. (2008). Formation of inorganic polymers (geopolymers) from 2:1 layer lattice aluminosilicates. J. Eur. Ceram. Soc..

[180] Yang T., Yao X., Zhang Z., Wang H. (2012). Mechanical property and structure of alkali-activated fly ash and slag blends. J. Sustain. Cem. Mater..

[181] Kumar S., Kumar R., Mehrotra S.P. (2010). Influence of granulated blast furnace slag on the reaction, structure and properties of fly ash based geopolymer. J. Mater. Sci..

[182] Shi C., Day R.L. (1999). Early strength development and hydration of alkali-activated blast furnace slag/fly ash blends. Adv. Cem. Res..

[183] Puertas F., Fernández-Jiménez A. (2003). Mineralogical and microstructural characterisation of alkali-activated fly ash/slag pastes. Cem. Concr. Compos..

[184] Sata V., Jaturapitakkul C., Kiattikomol K. (2007). Influence of pozzolan from various by-product materials on mechanical properties of high-strength concrete. Constr. Build. Mater..

[185] Tangchirapat W., Buranasing R., Jaturapitakkul C., Chindaprasirt P. (2008). Influence of rice husk–bark ash on mechanical properties of concrete containing high amount of recycled aggregates. Constr. Build. Mater..

[186] Rajaokarivony-Andriambololona Z., Thomassin J.H., Baillif P., Touray J.C. (1990). Experimental hydration of two synthetic glassy blast furnace slags in water and alkaline solutions (NaOH and KOH 0.1 N) at 40 °C: Structure, composition and origin of the hydrated layer. J. Mater. Sci..

[187] Teoreanu I. (1991). The interaction mechanism of blast-furnace slags with water. The role of the activating agents. IL Cem..

[188] Bernal S.A., Provis J.L., Walkley B., San Nicolas R., Gehman J.D., Brice D.G., Kilcullen A.R., Duxson P., Van Deventer J.S. (2013). Gel nanostructure in alkali-activated binders based on slag and fly ash, and effects of accelerated carbonation. Cem. Concr. Res..

[189] Singh G.B., Subramaniam K.V. (2017). Evaluation of sodium content and sodium hydroxide molarity on compressive strength of alkali activated low-calcium fly ash. Cem. Concr. Compos..

[190] Yang C., Gupta R. (2018). Prediction of the Compressive Strength from Resonant Frequency for Low-Calcium Fly Ash–Based Geopolymer Concrete. J. Mater. Civ. Eng..

[191] Helmuth R., Stark D., Diamond S., Moranville-Regourd M. (1993). Alkali-silica reactivity: An overview of research. Contract.

[192] Schmücker M., MacKenzie K. (2005). Microstructure of sodium polysialate siloxo geopolymer. Ceram. Int..

[193] Temuujin J., Van Riessen A., Williams R. (2009). Influence of calcium compounds on the mechanical properties of fly ash geopolymer pastes. J. Hazard. Mater..

[194] Temuujin J., Van Riessen A. (2009). Effect of fly ash preliminary calcination on the properties of geopolymer. J. Hazard. Mater..

[195] Yip C.K., Lukey G.C., Provis J.L., van Deventer J.S. (2008). Effect of calcium silicate sources on geopolymerisation. Cem. Concr. Res..

[196] He J., Jie Y., Zhang J., Yu Y., Zhang G. (2013). Synthesis and characterization of red mud and rice husk ash-based geopolymer composites. Cem. Concr. Compos..

[197] Islam A., Alengaram U.J., Jumaat M.Z., Bashar I.I. (2014). The development of compressive strength of ground granulated blast furnace slag-palm oil fuel ash-fly ash based geopolymer mortar. Mater. Des..

[198] Ranjbar N., Mehrali M., Behnia A., Alengaram U.J., Jumaat M.Z. (2014). Compressive strength and microstructural analysis of fly ash/palm oil fuel ash based geopolymer mortar. Mater. Des..

[199] Khater H. (2012). Effect of Calcium on Geopolymerization of Aluminosilicate Wastes. J. Mater. Civ. Eng..

[200] Nazari A., Bagheri A., Riahi S. (2011). Properties of geopolymer with seeded fly ash and rice husk bark ash. Mater. Sci. Eng. A.

[201] Qaidi S. (2022). Ultra-High-Performance Geopolymer Concrete. Part 3: Environmental Parameters.

[202] Qaidi S. (2022). Ultra-High-Performance Geopolymer Concrete. Part 4: Mix Design Methods.

[203] Qaidi S. (2022). Ultra-High-Performance Geopolymer Concrete. Part 5: Fresh Properties.

[204] Sata V., Sathonsaowaphak A., Chindaprasirt P. (2012). Resistance of lignite bottom ash geopolymer mortar to sulfate and sulfuric acid attack. Cem. Concr. Compos..

[205] Detphan S., Chindaprasirt P. (2009). Preparation of fly ash and rice husk ash geopolymer. Int. J. Miner. Metall. Mater..

[206] Duxson P., Provis J.L. (2008). Designing Precursors for Geopolymer Cements. J. Am. Ceram. Soc..

[207] Li Z., Xu G., Shi X. (2021). Reactivity of coal fly ash used in cementitious binder systems: A state-of-the-art overview. Fuel.

[208] Okoye F., Durgaprasad J., Singh N. (2015). Mechanical properties of alkali activated flyash/Kaolin based geopolymer concrete. Constr. Build. Mater..

[209] Van Chanh N., Trung B.D., Van Tuan D. Recent research geopolymer concrete. Proceedings of the 3rd ACF International Conference-ACF/VCA.

[210] Rangan B.V., Hardjito D., Wallah S.E., Sumajouw D.M. Studies on fly ash-based geopolymer concrete. Proceedings of the World Congress Geopolymer.

[211] Topark-Ngarm P., Chindaprasirt P., Sata V. (2015). Setting Time, Strength, and Bond of High-Calcium Fly Ash Geopolymer Concrete. J. Mater. Civ. Eng..

[212] Hardjito D., Wallah S.E., Sumajouw D.M.J., Rangan B.V. (2005). Fly Ash-Based Geopolymer Concrete. Aust. J. Struct. Eng..

[213] Sagoe-Crentsil K., Brown T., Taylor A. (2013). Drying shrinkage and creep performance of geopolymer concrete. J. Sustain. Cem.-Based Mater..

[214] Atmaja L., Fansuri H., Maharani A. (2011). Crystalline phase reactivity in the synthesis of fly ash-based geopolymer. Indones. J. Chem..

[215] Kupwade-Patil K., Allouche E.N. (2013). Impact of Alkali Silica Reaction on Fly Ash-Based Geopolymer Concrete. J. Mater. Civ. Eng..

[216] Tennakoon C., Shayan A., Sagoe-Crentsil K., Sanjayan J. Importance of reactive SiO_2_, AlO_3_ and Na_2_O in geopolymer formation. Proceedings of the 9th Austroads Bridge Conference.

[217] Thakur R.N., Ghosh S. (2009). Effect of mix composition on compressive strength and microstructure of fly ash based geopolymer composites. ARPN J. Eng. Appl. Sci..

[218] Bignozzi M.C., Manzi S., Natali M.E., Rickard W.D., van Riessen A. (2014). Room temperature alkali activation of fly ash: The effect of Na_2_O/SiO_2_ ratio. Constr. Build. Mater..

[219] Lloyd N., Rangan V. Geopolymer concrete with fly ash. Proceedings of the Second International Conference on Sustainable Construction Materials and Technologies, UWM Center for By-Products Utilization.

[220] Van Jaarsveld J.G.S., van Deventer J.S., Lukey G.C. (2002). The effect of composition and temperature on the properties of fly ash- and kaolinite-based geopolymers. Chem. Eng. J..

[221] Chotetanorm C., Chindaprasirt P., Sata V., Rukzon S., Sathonsaowaphak A. (2013). High-Calcium Bottom Ash Geopolymer: Sorptivity, Pore Size, and Resistance to Sodium Sulfate Attack. J. Mater. Civ. Eng..

[222] Qaidi S. (2022). Ultra-High-Performance Geopolymer Concrete. Part 6: Mechanical Properties.

[223] Qaidi S. (2022). Ultra-High-Performance Geopolymer Concrete. Part 7: Mechanical Performance Correlation.

[224] Qaidi S. (2022). Ultra-High-Performance Geopolymer Concrete. Part 8: Dynamic Behavior.

[225] Bernal S.A., Provis J.L. (2014). Durability of Alkali-Activated Materials: Progress and Perspectives. J. Am. Ceram. Soc..

[226] Phoo-Ngernkham T., Maegawa A., Mishima N., Hatanaka S., Chindaprasirt P. (2015). Effects of sodium hydroxide and sodium silicate solutions on compressive and shear bond strengths of FA–GBFS geopolymer. Constr. Build. Mater..

[227] Torres-Carrasco M., Puertas F. (2015). Waste glass in the geopolymer preparation. Mechanical and microstructural characterization. J. Clean. Prod..

[228] Ding Y.-C., Cheng T.-W., Dai Y.-S. (2017). Application of geopolymer paste for concrete repair. Struct. Concr..

[229] Phoo-Ngernkham T., Sata V., Hanjitsuwan S., Ridtirud C., Hatanaka S., Chindaprasirt P. (2015). High calcium fly ash geopolymer mortar containing Portland cement for use as repair material. Constr. Build. Mater..

[230] Demie S., Nuruddin M.F., Shafiq N. (2013). Effects of micro-structure characteristics of interfacial transition zone on the compressive strength of self-compacting geopolymer concrete. Constr. Build. Mater..

[231] Li Z., Chen R., Zhang L. (2013). Utilization of chitosan biopolymer to enhance fly ash-based geopolymer. J. Mater. Sci..

[232] Rattanasak U., Pankhet K., Chindaprasirt P. (2011). Effect of chemical admixtures on properties of high-calcium fly ash geopolymer. Int. J. Miner. Met. Mater..

[233] Nath S., Kumar S. (2013). Influence of iron making slags on strength and microstructure of fly ash geopolymer. Constr. Build. Mater..

[234] Zhang M., Zhao M., Zhang G., Mann D., Lumsden K., Tao M. (2016). Durability of red mud-fly ash based geopolymer and leaching behavior of heavy metals in sulfuric acid solutions and deionized water. Constr. Build. Mater..

[235] Adak D., Sarkar M., Mandal S. (2014). Effect of nano-silica on strength and durability of fly ash based geopolymer mortar. Constr. Build. Mater..

[236] Chindaprasirt P., Rattanasak U., Vongvoradit P., Jenjirapanya S. (2012). Thermal treatment and utilization of Al-rich waste in high calcium fly ash geopolymeric materials. Int. J. Miner. Met. Mater..

[237] Palomo A., Grutzeck M.W., Blanco M.T. (1999). Alkali-activated fly ashes: A cement for the future. Cem. Concr. Res..

[238] Andini S., Cioffi R., Colangelo F., Grieco T., Montagnaro F., Santoro L. (2008). Coal fly ash as raw material for the manufacture of geopolymer-based products. Waste Manag..

[239] Temuujin J., Williams R., van Riessen A. (2009). Effect of mechanical activation of fly ash on the properties of geopolymer cured at ambient temperature. J. Mater. Process. Technol..

[240] Chindaprasirt P., Rattanasak U., Taebuanhuad S. (2012). Resistance to acid and sulfate solutions of microwave-assisted high calcium fly ash geopolymer. Mater. Struct..

[241] Ridtirud C., Chindaprasirt P., Pimraksa K. (2011). Factors affecting the shrinkage of fly ash geopolymers. Int. J. Miner. Met. Mater..

[242] Giasuddin H.M., Sanjayan J.G., Ranjith P. (2013). Strength of geopolymer cured in saline water in ambient conditions. Fuel.

[243] Zelinkova M., Ondova M. (2015). Effect of Fly Ash Fineness on Sorption Properties of Geopolymers Based On Liquid Glass. Int. J. Civ. Environ. Eng..

[244] Shinde P., Patankar S., Sayyad A. (2018). Investigation on effects of fineness of flyash and alkaline ratio on mechanical properties of geopolymer concrete. Res. Eng. Struct. Mater..

[245] Jamkar S., Ghugal Y., Patankar S. (2013). Effect of fly ash fineness on workability and compressive strength of geopolymer concrete. Indian Concr. J..

[246] Firdaus, Yunus I., Rosidawani D. (2017). Contribution of Fineness Level of Fly Ash to the Compressive Strength of Geopolymer Mortar. MATEC Web Conf..

[247] Fernández-Jiménez A., Palomo A., Criado M. (2005). Microstructure development of alkali-activated fly ash cement: A descriptive model. Cem. Concr. Res..

[248] He P., Jia D., Wang M., Zhou Y. (2011). Thermal evolution and crystallization kinetics of potassium-based geopolymer. Ceram. Int..

[249] Qaidi S. (2022). Ultra-High-Performance Geopolymer Concrete. Part 9: Strain Hardening.

[250] Mohammed H., Ahmed S. (2020). Mechanical performance evaluation of concrete with waste coarse ceramic aggregate. Smart Cities—Opportunities and Challenges.

[251] Abdulkareem O.A., Ramli M. (2015). Optimization of Alkaline Activator Mixing and Curing Conditions for A fly Ash-Based Geopolymer Paste System. Mod. Appl. Sci..

[252] Villa C., Pecina E., Torres R., Gómez L. (2010). Geopolymer synthesis using alkaline activation of natural zeolite. Constr. Build. Mater..

[253] Noushini A., Aslani F., Castel A., Gilbert R.I., Uy B., Foster S. (2016). Compressive stress-strain model for low-calcium fly ash-based geopolymer and heat-cured Portland cement concrete. Cem. Concr. Compos..

[254] Karthik A., Sudalaimani K., Vijayakumar C., Saravanakumar S. (2018). Effect of bio-additives on physico-chemical properties of fly ash-ground granulated blast furnace slag based self-cured geopolymer mortars. J. Hazard. Mater..

[255] Alomayri T. (2019). Experimental study of the microstructural and mechanical properties of geopolymer paste with nano material (Al_2_O_3_). J. Build. Eng..

[256] Prabha V., Revathi V. (2019). Geopolymer Mortar Incorporating High Calcium Fly Ash and Silica Fume. Arch. Civ. Eng..

[257] Cai J., Pan J., Li X., Tan J., Li J. (2020). Electrical resistivity of fly ash and metakaolin based geopolymers. Constr. Build. Mater..

[258] Hajimohammadi A., Ngo T., Vongsvivut J. (2019). Interfacial chemistry of a fly ash geopolymer and aggregates. J. Clean. Prod..

[259] Fu Q., Xu W., Zhao X., Bu M., Yuan Q., Niu D. (2021). The microstructure and durability of fly ash-based geopolymer concrete: A review. Ceram. Int..

[260] Lloyd R.R., Provis J.L., van Deventer J.S.J. (2009). Microscopy and microanalysis of inorganic polymer cements. 1: Remnant fly ash particles. J. Mater. Sci..

[261] Qaidi S. (2022). Ultra-High-Performance Geopolymer Concrete. Part 11: Microstructural Properties.

[262] Parveen, Singhal D., Junaid M.T., Jindal B.B., Mehta A. (2018). Mechanical and microstructural properties of fly ash based geopolymer concrete incorporating alccofine at ambient curing. Constr. Build. Mater..

[263] Aligizaki K.K. (2005). Pore Structure of Cement-Based Materials: Testing, Interpretation and Requirements.

[264] Puertas F., Palacios M., Manzano H., Dolado J.S., Rico A., Rodríguez J. (2011). A model for the C-A-S-H gel formed in alkali-activated slag cements. J. Eur. Ceram. Soc..

[265] Najm H.M., Ahmad S., Khan R.A. (2022). Mechanical and Microstructural Analysis of Waste Ceramic Optimal Concrete Reinforced by Hybrid Fibers Materials: A Comprehensive Study. J. Archit. Environ. Struct. Eng. Res..

[266] Najm H.M., Nanayakkara O., Ahmad M., Sabri Sabri M.M. (2022). Mechanical Properties, Crack Width, and Propagation of Waste Ceramic Concrete Subjected to Elevated Temperatures: A Comprehensive Study. Materials.

[267] Najm H.M., Nanayakkara O., Ahmad M., Sabri Sabri M.M. (2022). Colour Change of Sustainable Concrete Containing Waste Ceramic and Hybrid Fibre: Effect of Temperature. Materials.

[268] Najm H.M., Ahmad S. (2021). Effect of elevated temperatures exposure on the mechanical properties of waste ceramic concrete reinforced with hybrid fibers materials. Sigma J. Eng. Nat. Sci..

[269] Najm H.M., Ahmad S. (2022). The Use of Waste Ceramic Optimal Concrete for A Cleaner and Sustainable Environment—A Case Study of Mechanical Properties. Civ. Environ. Eng. Rep..

[270] Najm H.M., Ahmad S., Submitter Y. (2021). Artificial Neural Networks for Evaluation & Prediction of the Mechanical Properties of Waste Ceramic Optimal Concrete Exposed to Elevated Temperature. SSRN.

